# CHCHD2 P14L, found in amyotrophic lateral sclerosis, exhibits cytoplasmic mislocalization and alters Ca^2+^ homeostasis

**DOI:** 10.1093/pnasnexus/pgae319

**Published:** 2024-07-30

**Authors:** Aya Ikeda, Hongrui Meng, Daisuke Taniguchi, Muneyo Mio, Manabu Funayama, Kenya Nishioka, Mari Yoshida, Yuanzhe Li, Hiroyo Yoshino, Tsuyoshi Inoshita, Kahori Shiba-Fukushima, Yohei Okubo, Takashi Sakurai, Taku Amo, Ikuko Aiba, Yufuko Saito, Yuko Saito, Shigeo Murayama, Naoki Atsuta, Ryoichi Nakamura, Genki Tohnai, Yuishin Izumi, Mitsuya Morita, Asako Tamura, Osamu Kano, Masaya Oda, Satoshi Kuwabara, Toru Yamashita, Jun Sone, Ryuji Kaji, Gen Sobue, Yuzuru Imai, Nobutaka Hattori

**Affiliations:** Department of Neurology, Juntendo University Graduate School of Medicine, Bunkyo-ku, Tokyo 113-8421, Japan; Research Institute for Diseases of Old Age, Juntendo University Graduate School of Medicine, Bunkyo-ku, Tokyo 113-8421, Japan; Department of Research for Parkinson's Disease, Juntendo University Graduate School of Medicine, Bunkyo-ku, Tokyo 113-8421, Japan; Department of Neurology, Juntendo University Graduate School of Medicine, Bunkyo-ku, Tokyo 113-8421, Japan; Department of Neurology, Juntendo University Graduate School of Medicine, Bunkyo-ku, Tokyo 113-8421, Japan; Department of Neurology, Juntendo University Graduate School of Medicine, Bunkyo-ku, Tokyo 113-8421, Japan; Research Institute for Diseases of Old Age, Juntendo University Graduate School of Medicine, Bunkyo-ku, Tokyo 113-8421, Japan; Center for Genomic and Regenerative Medicine, Juntendo University Graduate School of Medicine, Bunkyo-ku, Tokyo 113-8421, Japan; Department of Neurology, Juntendo University Graduate School of Medicine, Bunkyo-ku, Tokyo 113-8421, Japan; Department of Neuropathology, Institute for Medical Science of Aging, Aichi Medical University, Nagakute, Aichi 480-1195, Japan; Department of Neurology, Juntendo University Graduate School of Medicine, Bunkyo-ku, Tokyo 113-8421, Japan; Research Institute for Diseases of Old Age, Juntendo University Graduate School of Medicine, Bunkyo-ku, Tokyo 113-8421, Japan; Department of Neurology, Juntendo University Graduate School of Medicine, Bunkyo-ku, Tokyo 113-8421, Japan; Department of Drug Development for Parkinson's Disease, Juntendo University Graduate School of Medicine, Bunkyo-ku, Tokyo 113-8421, Japan; Department of Cellular and Molecular Pharmacology, Juntendo University Graduate School of Medicine, Bunkyo-ku, Tokyo 113-8421, Japan; Department of Cellular and Molecular Pharmacology, Juntendo University Graduate School of Medicine, Bunkyo-ku, Tokyo 113-8421, Japan; Department of Applied Chemistry, National Defense Academy, Yokosuka, Kanagawa 239-8686, Japan; Department of Neurology, NHO Higashinagoya National Hospital, Meito-ku, Nagoya, Aichi 465-8620, Japan; Department of Neurology, NHO Higashinagoya National Hospital, Meito-ku, Nagoya, Aichi 465-8620, Japan; Brain Bank for Aging Research (Department of Neuropathology), Tokyo Metropolitan Institute for Geriatrics and Gerontology, Tokyo 173-0015, Japan; Brain Bank for Aging Research (Department of Neuropathology), Tokyo Metropolitan Institute for Geriatrics and Gerontology, Tokyo 173-0015, Japan; Brain Bank for Neurodevelopmental, Neurological and Psychiatric Disorders, United Graduate School of Child Development, Osaka University, Osaka 565-0871, Japan; Department of Neurology, Aichi Medical University School of Medicine, Nagakute, Aichi 480-1195, Japan; Department of Neurology, Aichi Medical University School of Medicine, Nagakute, Aichi 480-1195, Japan; Division of ALS Research, Aichi Medical University School of Medicine, Nagakute, Aichi 480-1195, Japan; Department of Neurology, Tokushima University Graduate School of Biomedical Sciences, Tokushima 770-8503, Japan; Division of Neurology, Department of Internal Medicine, Jichi Medical University, Shimotsuke, Tochigi 329-0498, Japan; Department of Neurology, Mie University Graduate School of Medicine, Tsu, Mie 514-8507, Japan; Department of Neurology, Toho University Faculty of Medicine, Ota-ku, Tokyo 143-8541, Japan; Department of Neurology, Vihara Hananosato Hospital, Miyoshi, Hiroshima 728-0001, Japan; Department of Neurology, Graduate School of Medicine, Chiba University, Chuo-ku, Chiba 260-8670, Japan; Department of Neurology, Okayama University Graduate School of Medicine, Kita-ku, Okayama 700-8558, Japan; Department of Neuropathology, Institute for Medical Science of Aging, Aichi Medical University, Nagakute, Aichi 480-1195, Japan; Department of Clinical Neuroscience, Tokushima University, Tokushima 770-8503, Japan; Aichi Medical University, Nagakute, Aichi 480-1195, Japan; Department of Neurology, Juntendo University Graduate School of Medicine, Bunkyo-ku, Tokyo 113-8421, Japan; Department of Research for Parkinson's Disease, Juntendo University Graduate School of Medicine, Bunkyo-ku, Tokyo 113-8421, Japan; Department of Neurology, Juntendo University Graduate School of Medicine, Bunkyo-ku, Tokyo 113-8421, Japan; Department of Research for Parkinson's Disease, Juntendo University Graduate School of Medicine, Bunkyo-ku, Tokyo 113-8421, Japan; Department of Drug Development for Parkinson's Disease, Juntendo University Graduate School of Medicine, Bunkyo-ku, Tokyo 113-8421, Japan; Neurodegenerative Disorders Collaborative Laboratory, RIKEN Center for Brain Science, Wako, Saitama 351-0198, Japan

**Keywords:** amyotrophic lateral sclerosis, Parkinson's disease, mitochondria, TDP-43, Ca^2+^ dynamics

## Abstract

CHCHD2 and CHCHD10, linked to Parkinson's disease and amyotrophic lateral sclerosis-frontotemporal dementia (ALS), respectively, are mitochondrial intermembrane proteins that form a heterodimer. This study aimed to investigate the impact of the CHCHD2 P14L variant, implicated in ALS, on mitochondrial function and its subsequent effects on cellular homeostasis. The missense variant of CHCHD2, P14L, found in a cohort of patients with ALS, mislocalized CHCHD2 to the cytoplasm, leaving CHCHD10 in the mitochondria. *Drosophila* lacking the *CHCHD2* ortholog exhibited mitochondrial degeneration. In contrast, human CHCHD2 P14L, but not wild-type human CHCHD2, failed to suppress this degeneration, suggesting that P14L is a pathogenic variant. The mitochondrial Ca^2+^ buffering capacity was reduced in *Drosophila* neurons expressing human CHCHD2 P14L. The altered Ca^2+^-buffering phenotype was also observed in cultured human neuroblastoma SH-SY5Y cells expressing CHCHD2 P14L. In these cells, transient elevation of cytoplasmic Ca^2+^ facilitated the activation of calpain and caspase-3, accompanied by the processing and insolubilization of TDP-43. These observations suggest that CHCHD2 P14L causes abnormal Ca^2+^ dynamics and TDP-43 aggregation, reflecting the pathophysiology of ALS.

**Significance Statement**

Mutations in CHCHD2 and CHCHD10 cause Parkinson's disease (PD) and amyotrophic lateral sclerosis-frontotemporal dementia (ALS-FTD), respectively. While both CHCHD2 and CHCHD10 are necessary for mitochondrial function, the reasons for mutations in these genes causing different neurodegenerative diseases remain unclear. This study showed distinct behavior between the ALS-associated CHCHD2 P14L variant and the PD-associated CHCHD2 T61I. CHCHD2 T61I insolubilized in the mitochondria together with CHCHD10, whereas CHCHD2 P14L leaked from the mitochondria into the cytoplasm. Disease-associated stress accelerated CHCHD2 P14L leakage into the cytoplasm, leading to cytochrome c release and caspase activation. The reduced mitochondrial Ca^2+^-buffering capacity by CHCHD2 P14L promoted TDP-43 fragmentation and insolubilization by caspases and calpain. These effects of CHCHD2 P14L may promote motor neuron death in ALS.

## Introduction

*CHCHD2* and *CHCD10*, genes associated with Parkinson's disease (PD) and amyotrophic lateral sclerosis-frontotemporal dementia (ALS-FTD), respectively, are localized within the mitochondrial intermembrane space. These genes are involved in the regulation of mitochondrial respiratory chain complexes and maintenance of the structure of cristae (1–4). *CHCHD2* and *CHCHD10* are thought to be evolutionarily paralogous and yeast (5), *Caenorhabditis elegans* (6), and *Drosophila* (7) possess a prototype gene. CHCHD2 and CHCHD10 are highly similar at the amino acid level (8), and thus far, no differences in their essential cellular functions have been identified (2). Therefore, understanding why *CHCHD2* and *CHCHD10* mutations result in different neurodegenerative diseases is crucial.

We previously reported the phenotypic analysis of *CHCHD2/CHCHD10* ortholog *CG5010* (hereafter referred to as *dCHCHD2*) knockout flies and found that the loss of *dCHCHD2* produces large amounts of reactive oxygen species (ROS) and leads to progressive destruction of cristae structures (7). Identification of CHCHD2-binding proteins in cultured human cells revealed that CHCHD2 stabilizes cytochrome c (cyt C) in the respiratory chain complex. This facilitates electron transfer from respiratory chain complex III to IV, thereby suppressing ROS generation resulting from electron leakage (7). In contrast, the loss of CHCHD2 and the expression of PD-associated mutants promote the release of cyt C from the mitochondria and subsequent activation of caspase-3/-7 by anticancer drugs and oxidative stress (7).

Pathological analysis revealed that a patient with PD carrying CHCHD2 T61I presented widespread α-synuclein aggregation (9). This α-synuclein aggregation and accumulation was reproduced in *Drosophila* and iPS-derived dopaminergic neurons from another patient with PD carrying CHCHD2 T61I (9). CHCHD2 T61I transgenic and knock-in mice also showed accumulation of insoluble α-synuclein and phospho-α-synuclein in the brain (10, 11). Additionally, various proteins, including mitochondrial proteins, demonstrate increased insolubilization in the brains of CHCHD2 T61I transgenic mice (10). These findings suggest that CHCHD2 mutations widely affect intracellular proteostasis.

TDP-43, a heterogeneous nuclear ribonucleoprotein, shuttles between the nucleus and cytoplasm and is thought to exert multiple functions, including the regulation of RNA splicing, transport, and stabilization (12–14). Mislocalization of TDP-43 to the cytoplasm and nuclear loss of TDP-43 are associated with neurodegeneration (15). TDP-43 is presumed to maintain its function through autoregulation and tightly regulated nuclear-cytoplasmic transport. Disruption of this regulatory mechanism leads to neurodegeneration (15).

In ALS-FTD, numerous RNA-binding proteins, including TDP-43, C9orf72, and FUS, accumulate in affected regions. These RNA-binding proteins can cause liquid–liquid phase separation (LLPS) (16–18). LLPS is thought to transiently form nonmembrane organelles, contributing to efficient biochemical responses and unique functions within the droplets (19). However, persistent cellular stress or mutations that affect the 3D structure of low-complexity domains could cause droplet ageing and fibrillization over time (20, 21). Mutations in TDP-43 can also cause ALS-FTD, and most disease-associated mutations reside in its low-complexity domains at the glycine-rich C-terminus (15). Aggregation of TDP-43 has been observed in affected neurons and glia in ALS-FTD and other neurodegenerative diseases, including Alzheimer's disease (22–25), PD (26–28), Huntington's disease (29), and Perry's syndrome (29, 30). These diseases with TDP-43 inclusion body pathology constitute a full spectrum of TDP-43 proteinopathies, characterized by the presence of hyperphosphorylated, fragmented, and aggregated TDP-43 (15, 22, 31–36). However, firm evidence of TDP-43 accumulation in ALS-FTD linked to *CHCHD10* mutations is lacking. Nonetheless, TDP-43 accumulation has been reported in ALS-FTD-associated *CHCHD10* mutant cells and animal models (2).

Various reports have explored the relationship between mitochondria and TDP-43. Oxidative stress caused by mitochondrial degeneration has been suggested as a possible mechanism for TDP-43 aggregation (37). Additionally, TDP-43 toxicity in upper motor neurons has been reported to be reduced when the disruption of mitochondrial and endoplasmic reticulum (ER) integrity by misfolded SOD1 is improved by drug administration (38). In contrast, some reports suggest that TDP-43 physiologically regulates mitochondrial function, and its disruption leads to mitochondrial degeneration. Disease-associated TDP-43 mutations promote mitochondrial localization, resulting in the inhibition of mRNA translation of respiratory complex I subunits (39). The aggregation of TDP-43 inhibits the local translation of nuclear-encoded mitochondrial proteins at neuronal terminals (14). TDP-43 induces mitochondrial unfolded protein stress (mUPR), and mitochondrial protease LonP1 is upregulated in response to mUPR to remove TDP-43 from the mitochondria (40). This suggests a vicious cycle wherein mitochondrial dysfunction leads to the aggregation of TDP-43, which in turn threatens mitochondrial function.

Based on the similarities between CHCHD2 and CHCHD10, we hypothesized that mutations in *CHCHD2* may cause ALS. Genetic screening of a Japanese ALS cohort revealed two rare variants, −8T > G and c.41C > T (p.P14L). This study aimed to investigate the impact of the CHCHD2 P14L variant on mitochondrial function and its subsequent effects on cellular homeostasis. We analyzed the effect of the P14L missense variant on CHCHD2 function in cultured human cells and *Drosophila* models. This study found that CHCHD2 P14L tends to leak into the cytoplasm, reducing the mitochondrial Ca^2+^ buffering capacity and causing a transient increase in cytosolic Ca^2+^. Elevated cytosolic Ca^2+^ levels promote cyt C release and caspase/calpain activation, followed by fragmentation and insolubilization of TDP-43. Our study suggests that altered Ca^2+^ dynamics induced by CHCHD2 P14L cause the development of ALS.

## Results

### The ALS-associated CHCHD2 P14L variant is mislocalized in the cytoplasm

**Fig. 1.**
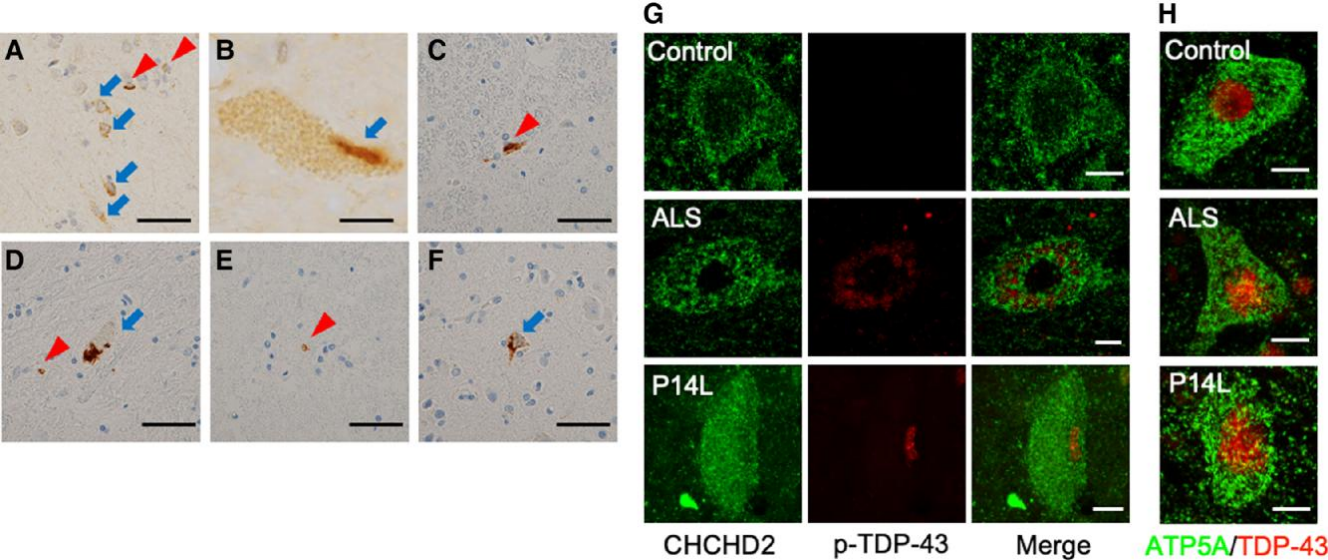
TDP-43 pathology in ALS cases with CHCHD2 variants. A) Neuronal cytoplasmic inclusions (NCIs, arrows) and glial cytoplasmic inclusions (GCIs, arrowheads) in the precentral gyrus. B) Skein-like inclusion (arrow) in the anterior horn of the cervical spinal cord. C) GCI (arrowhead) in the anterior horn of the cervical spinal cord. D) NCI (arrow) and GCI (arrowhead) in the anterior horn of the thoracic spinal cord. E) GCI (arrowhead) in the anterior horn of the lumbar spinal cord. F) NCI (arrow) in the precentral gyrus. A, B) *CHCHD2* −8T > G. C–F) CHCHD2 P14L. G, H) Subcellular localization of CHCHD2 and phospho-S409/410 TDP-43 (G) and ATP5A and TDP-43 (H) in motor neurons of the anterior horn of the spinal cord. ATP5A served as a mitochondrial marker. Scale bars, 40 µm (A, C–F) and 10 µm (B, G, H). Related data are shown in Fig. S2.

The screening of 944 patients with ALS in the Japanese Consortium for ALS Research (JaCALS) cohort identified two *CHCHD2* variants, −8T > G and c.41C > T (p.P14L) (SI Appendix, Fig. S1A). The patient with −8T > G also had an additional unknown variant, c.628A > G (p.I210 V), in ALS-related *senataxin* (*SETX*) gene (41). c.41C > T allele frequency in the JaCALS cohort was higher than that in both the Japanese and East Asian general populations (SI Appendix, Table S1). On the contrary, the allele frequency of −8T > G in the JaCALS cohort did not differ from that in the general East Asian population (SI Appendix, Table S1). Typical neuronal and glial TDP-43 inclusions were detected in the precentral gyrus and spinal cord of patients harboring these variants (Fig. 1A–F). As previously reported, in normal neurons, TDP-43 was localized to the nucleus and was not phosphorylated at Ser409/410 (Fig. 1 G and H and SI Appendix, Fig. S2). In contrast, cytoplasmic inclusions of TDP-43 were phosphorylated in patients with CHCHD2 variants or sporadic ALS (Fig. 1G and H and SI Appendix, Fig. S2).

**Fig. 2.**
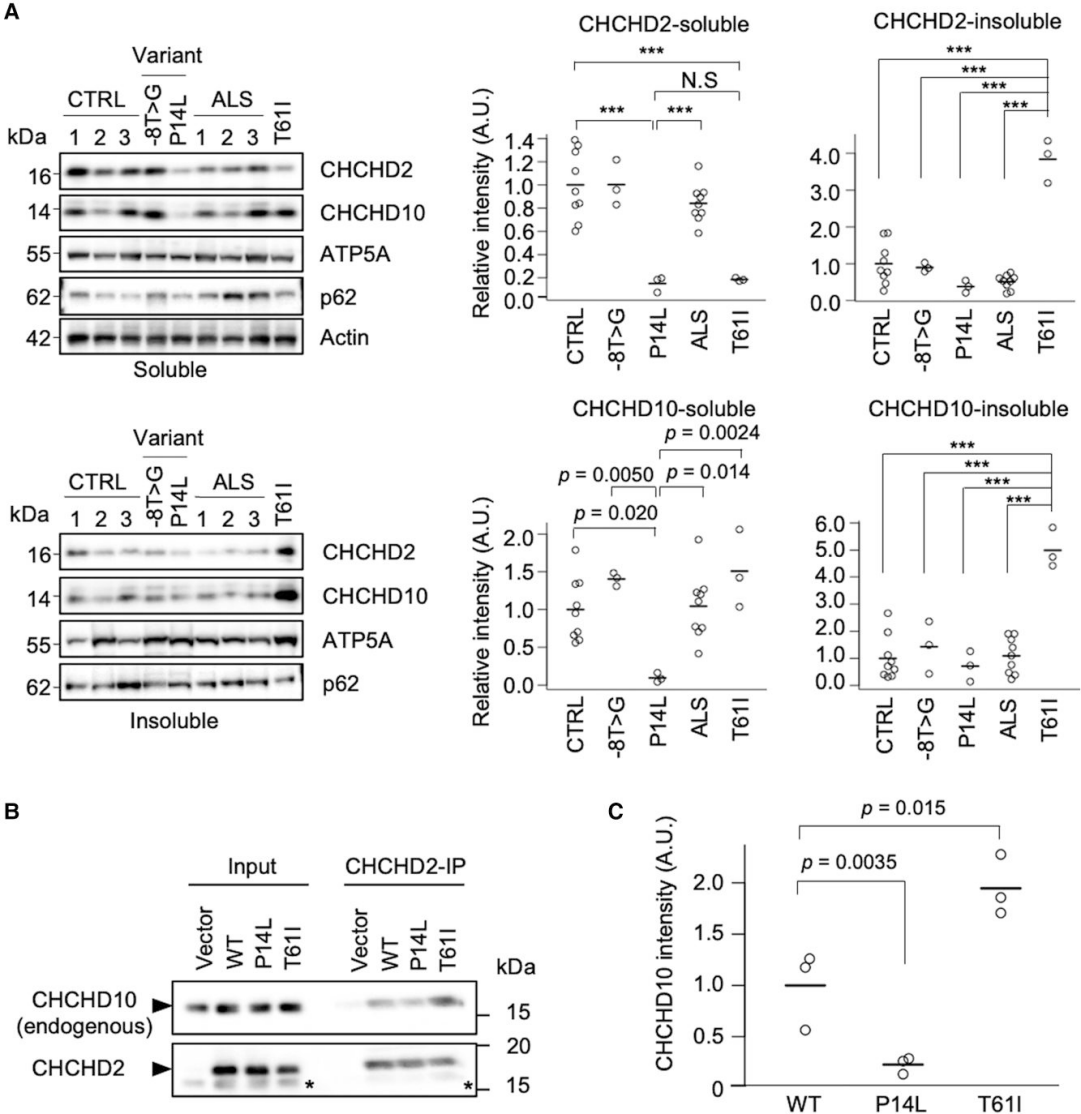
CHCHD2 P14L shows less binding to CHCHD10. A) Quantitative western blot of 1% sarkosyl-soluble and insoluble fractions from brain autopsies of three patients with sporadic ALS (ALS) and patients with ALS carrying CHCHD2 −8T > G and P14L, along with a patient with PD carrying CHCHD2 T61I. ATP5A and actin served as mitochondria and total protein loading controls, respectively. Comparison was determined by Tukey–Kramer's test. ***P < 0.001. A.U., arbitrary units. B, C) CHCHD2 P14L shows weaker binding affinity for CHCHD10. B) Lysate of CHCHD2^−/−^ SH-SY5Y cells harboring CHCHD2 variants or a mock vector was subjected to immunoprecipitation using an anti-CHCHD2 antibody. Asterisks indicate the remaining CHCHD10 signals because the western blot using an anti-CHCHD2 antibody was performed sequentially after that using an anti-CHCHD10 antibody. C) The band intensities of CHCHD10 co-precipitated with CHCHD2, normalized with those of CHCHD2, were graphed. Comparison was determined by Tukey–Kramer's test from three biological replicates.

Biochemical analysis of the autopsied brain tissue with PD-associated CHCHD2 T61I revealed the insolubility of CHCHD2 and CHCHD10, as previously reported (lower panel in Fig. 2A) (9). In contrast, the expression of CHCHD2 in the brain tissue with the CHCHD2 −8T > G variant was comparable to that in normal controls (upper panel in Fig. 2A). CHCHD2 and CHCHD10 appeared to have reduced protein expression levels in brain tissue with the CHCHD2 P14L variant (upper panel in Fig. 2A). Consistent with this observation, the expression of *CHCHD2* transcripts was decreased in patients with P14L (SI Appendix, Fig. S1B). However, *CHCHD10* transcripts were not downregulated, suggesting changes in protein expression (SI Appendix, Fig. S1B). Both *CHCHD2* and *CHCHD10* transcripts were upregulated in patients with sporadic ALS (SI Appendix, Fig. S1B). Although the upregulation of *CHCHD2* and *CHCHD10* is not reflected at the protein levels, previous observations that CHCHD2 and CHCHD10 are elevated in cultured cells and *Drosophila* under various types of mitochondrial stress provide evidence that mitochondrial stress is also involved in sporadic ALS (42, 43). The expression levels of ATP5A and p62 in the brains with ALS-associated *CHCHD2* variants did not differ from those in normal controls (Fig. 2A).

We generated *CHCHD2* knockout SH-SY5Y cells and retrovirally reintroduced CHCHD2 variants or a mock vector (SI Appendix, Fig. S1C). As −8T > G is not likely to be a pathogenic variant, we characterized the protein properties of P14L compared with those of the wild-type (WT) and T61I. The expression of each CHCHD2 was found to be at endogenous levels (SI Appendix, Fig. S1C). CHCHD2 and CHCHD10 form a heterodimer (4). CHCHD2 T61I bound more strongly to CHCHD10 than the WT in these cells (Fig. 2B and C). In contrast, P14L exhibited a weaker interaction with CHCHD10 (Fig. 2B and C).

**Fig. 3.**
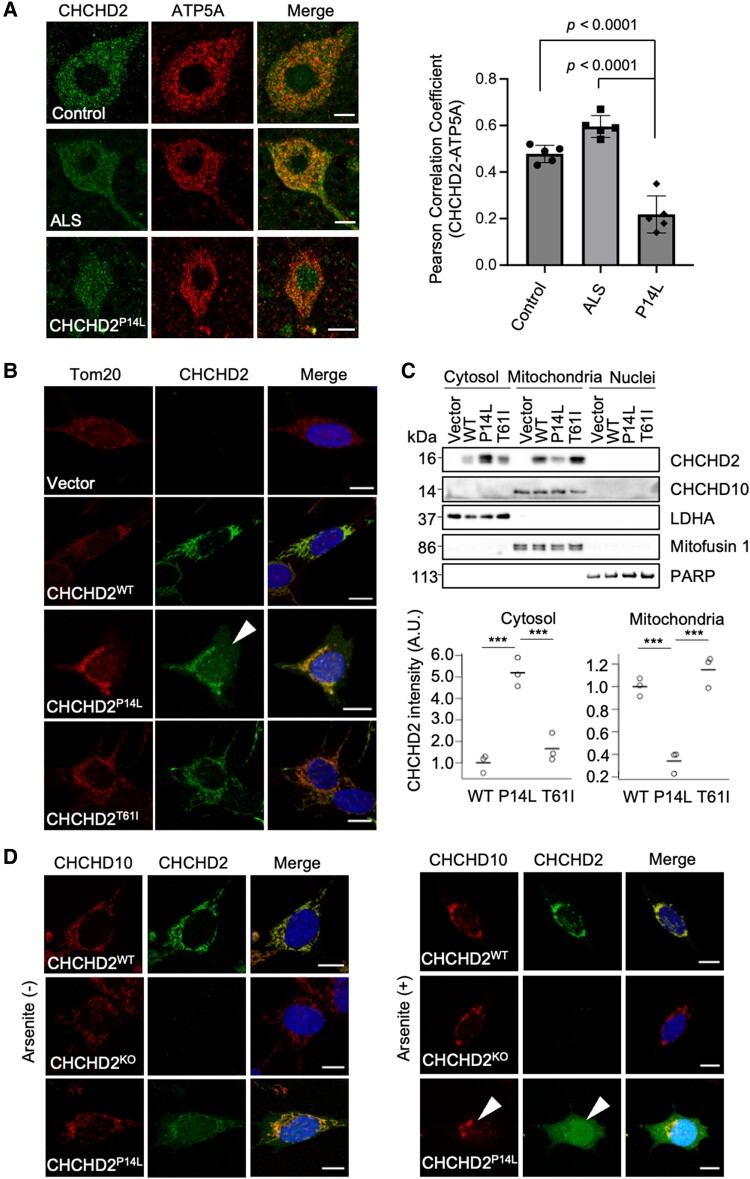
CHCHD2 P14L is released from mitochondria. A) Subcellular localization of CHCHD2 and ATP5A in motor neurons of the anterior horn of the spinal cord. Scale bars, 10 µm. The graph shows the Pearson's correlation coefficients based on the pathological staining results as shown on the left. Comparison was determined by Tukey–Kramer's test. *n* = 5 cells in each group from autopsies of two controls, two patients with sporadic ALS (ALS), and one patient with ALS carrying the CHCHD2 P14L variant. B) Dissociation of CHCHD2 P14L, but not WT or T61I, from the mitochondria to the cytoplasm. An arrowhead indicates the cytoplasmic CHCHD2. CHCHD2, Tom20, and the nuclei (blue) were visualized in each SH-SY5Y cell line. Tom20 served as a mitochondrial marker. C) Biochemical fractionation of CHCHD2 in SH-SY5Y cell lines. LDHA, Mitofusin 1, and PARP served as cytoplasmic, mitochondrial, and nucleic markers, respectively. Graphs indicate the quantified CHCHD2 signals in each fraction. ***P < 0.001 by Tukey–Kramer's test from three biological replicates. A.U., arbitrary units. D) CHCHD2 P14L does not translocate CHCHD10 to the cytoplasm. CHCHD2^−/−^ SH-SY5Y cells harboring a mock vector (KO), CHCHD2 WT, or P14L were treated with or without 0.5 mM arsenite for 1 h. CHCHD2 and CHCHD10 were visualized with specific antibodies. Arrowheads indicate the lack of colocalization of CHCHD2 and CHCH10. Scale bars, 10 μm.

To further examine whether the molecular behavior of P14L associated with ALS differ from those of PD-associated T61I, we analyzed the subcellular localization of CHCHD2 in neurons from patients with ALS carrying CHCHD2 P14L. Neurons harboring CHCHD2 P14L showed mislocalization of CHCHD2 from the mitochondria (Fig. 3A). Furthermore, CHCHD10 showed reduced colocalization with CHCHD2 (SI Appendix, Fig. S3). Next, we confirmed this phenomenon in *CHCHD2^−/−^* SH-SY5Y cells expressing each CHCHD2 variant. The CHCHD2 WT and T61I were mainly localized to the mitochondria (Fig. 3B and C and SI Appendix, Fig. S4A). In contrast, CHCHD2 P14L was partially localized to the cytoplasm and mitochondria (Fig. 3B and C and SI Appendix, Fig. S4A). Arsenite treatment promoted the cytoplasmic localization of P14L, but not of WT (Fig. 3D and SI Appendix, Fig. S4B). Importantly, CHCHD10 was not associated with the cytoplasmic localization of P14L induced by arsenite treatment and remained aggregated in the mitochondria (Fig. 3D and SI Appendix, Fig. S4B). However, prominent aggregation of TDP-43 or cytoplasmic translocation of TDP-43 was not observed under this condition (SI Appendix, Fig. S4C). Next, we tested whether the cytoplasmic localization of P14L was caused by impaired mitochondrial transport during biosynthesis or by translocation to the cytoplasm once it had been transported to the mitochondria. Mitochondria isolated from *CHCHD2^−/−^* SH-SY5Y cells were incubated with CHCHD2 biosynthesized in vitro, and mitochondrial transport was monitored over time. The results showed that CHCHD2 P14L exhibited efficient mitochondrial transport, comparable to that of the WT or T61I (SI Appendix, Fig. S5). These results indicate that CHCHD2 P14L leaks into the cytoplasm once it is transported to the mitochondria. The observation that arsenite treatment enhances P14L leakage into the cytoplasm also supports this hypothesis.

### CHCHD2 genetically interacts with TDP-43

**Fig. 4.**
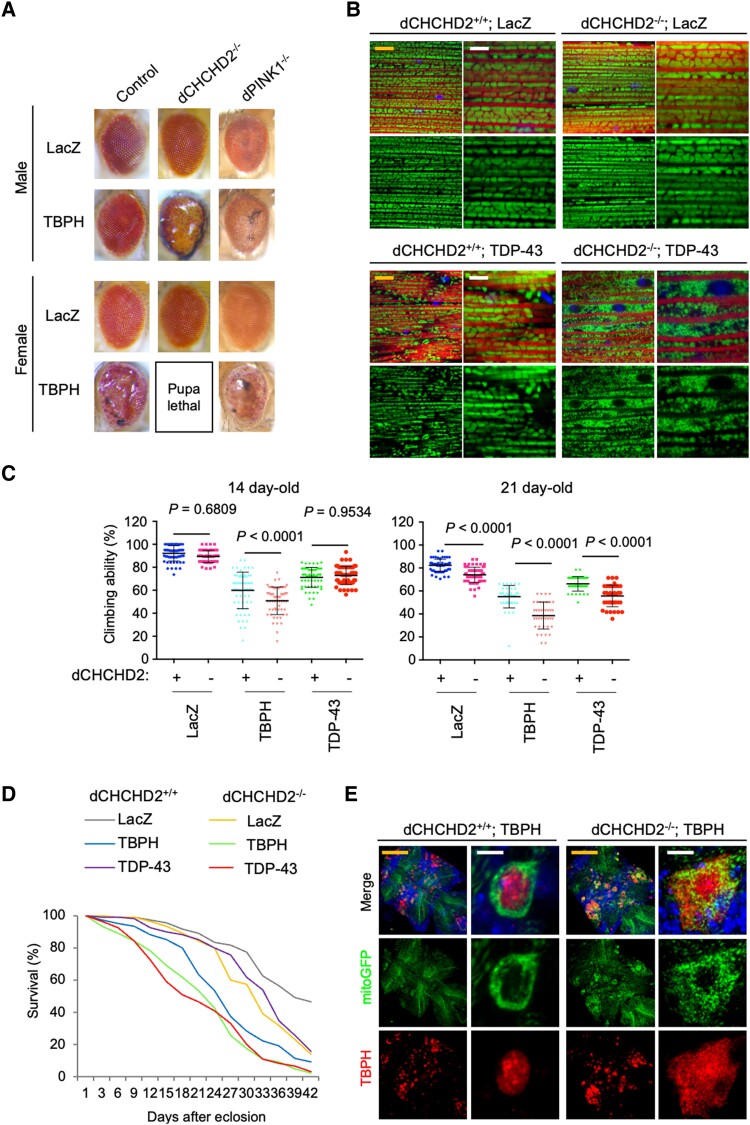
Genetic interaction between CHCHD2 and TDP-43. A) Loss of CHCHD2 enhances TDP-43 toxicity. TBPH or LacZ was expressed in the eyes of the normal control, *dCHCHD2-deficient,* or *dPINK1*-deficient flies. LacZ served as a mock control. B) Expression of human TDP-43 causes mitochondrial fragmentation and nuclear swelling in the absence of dCHCHD2. Mitochondria (green), myofibrils (red), and nuclei (blue) were visualized by mitoGFP, phalloidin-TRITC, and DAPI, respectively. Orange and white scale bars, 5 µm and 2 µm, respectively. C) The motor ability of *dCHCHD2^+/+^* or *dCHCHD2^−/−^* flies expressing LacZ, TBPH, or human TDP-43. The transgenes were driven by the motor neuron-specific driver *D42-GAL4*. Fourteen-day-old (20 trials with 43–57 flies in three independent vials) and 21-day-old flies (20 trials with 26–54 flies in 2–3 independent vials) were analyzed and compared using Tukey–Kramer's test. D) Human TDP-43 or TBPH exacerbates the shortened lifespan phenotype of *dCHCHD2^−/−^* flies. *P* < 0.0001, *dCHCHD2^−/−^*; *TBPH* or *dCHCHD2^−/−^*; *TDP-43* vs. *dCHCHD2^−/−^*; *LacZ* by log-rank test. *n* = 120 in each group. Transgenes were driven by *D42-GAL4*. E) TBPH is localized outside the nuclei of motor neurons by dCHCHD2 loss. MitoGFP and TBPH-FLAG (red) were expressed by *D42-GAL4* in motor neurons of the thoracic ganglion. TBPH-FLAG and the nuclei were stained with anti-FLAG and DAPI (blue), respectively. The left and right columns show images of the thoracic ganglion and magnified images of the motor neuron cell bodies in the thoracic ganglion, respectively. Motor neuron mitochondria of *dCHCHD2^−/−^* flies were prominently fragmented. Orange and white scale bars, 100 µm and 10 µm, respectively.

To investigate whether the loss or mutations of CHCHD2 caused TDP-43 accumulation, we examined the genetic interaction between dCHCHD2 and the *Drosophila* TDP-43 ortholog TBPH in *Drosophila*. TBPH overexpression caused a rough-eye phenotype in female but not in male animals (Fig. 4A) (44). Under these conditions, the loss of dCHCHD2 resulted in severe rough-eye phenotypes in males and lethality in females (Fig. 4A). Loss of dPINK1, another mitochondria-associated PD gene, also exacerbated eye degeneration caused by TBPH overexpression, but its phenotype was milder than that of dCHCHD2 (Fig. 4A).

Overexpression of human TDP-43 in muscles caused abnormal wing posture in *dCHCHD2^−/−^* flies, whereas the effect was milder in *dCHCHD2^+/+^* flies (SI Appendix, Fig. S6A). These phenotypes appeared to originate from muscular degeneration. Consistent with the wing phenotypes, mild disorganization of myofibrils was observed following TDP-43 expression in *dCHCHD2^+/+^* flies. In contrast, the expression of TDP-43 in the absence of dCHCHD2 resulted in much more severe muscle degeneration, where mitochondrial fragmentation and nuclear swelling were observed (Fig. 4B).

The loss of dCHCHD2 decreased motor ability in 21-day-old flies, as previously reported (7). The expression of TBPH or TDP-43 in motor neurons affected motor ability, which was enhanced by the loss of dCHCHD2 in an age-dependent manner (Fig. 4C). In the same setting, the short-lifespan phenotype of *dCHCHD2^−/−^* flies was exacerbated by the expression of TBPH or TDP-43 in motor neurons (Fig. 4D).

TBPH was mainly localized in the nuclei of motor neurons in the thoracic ganglion, as well as in the nuclei of muscle cells in normal adult flies, as previously reported (Fis. 4E and SI Appendix, Fig. S7) (45, 46). Similar to the TDP-43 pathology in ALS, cytoplasmic translocation of TBPH was detected in the motor neurons of *dCHCHD2^−/−^* flies (Fig. 4E) (16). Furthermore, mitochondrial fragmentation was also observed, as seen in muscle cells (Fig. 4E vs. B).

### Loss of CHCHD2 promotes mitochondrial localization of TDP-43

Although TDP-43 is a nuclear protein, mitochondrial translocation of TDP-43 in motor neurons has been reported in patients with sporadic ALS or FTLD, as well as in those with ALS-associated TDP-43 mutants (39). Consistent with these reports, nuclear TBPH was translocated to the mitochondria or perimitochondrial regions by loss of dCHCHD2 (SI Appendix, Fig. S8A) (39). Furthermore, the loss of CHCHD2 promoted the aggregation of nuclear or perinuclear TBPH, in which polyubiquitin (polyUb) and Ref(2)P accumulated (SI Appendix, Fig. S9). Immunoelectron microscopy confirmed that endogenous TBPH immunosignals were mainly localized in the nucleus and were largely absent in the mitochondria of normal muscle cells (SI Appendix, Fig. S8B–E). In contrast, TBPH signals were frequently observed in the degenerated mitochondria of *dCHCHD2^−/−^* flies (SI Appendix, Fig. S8F–H).

### ALS-associated CHCHD2 P14L does not rescue *dCHCHD2^−/−^* phenotypes

**Fig. 5.**
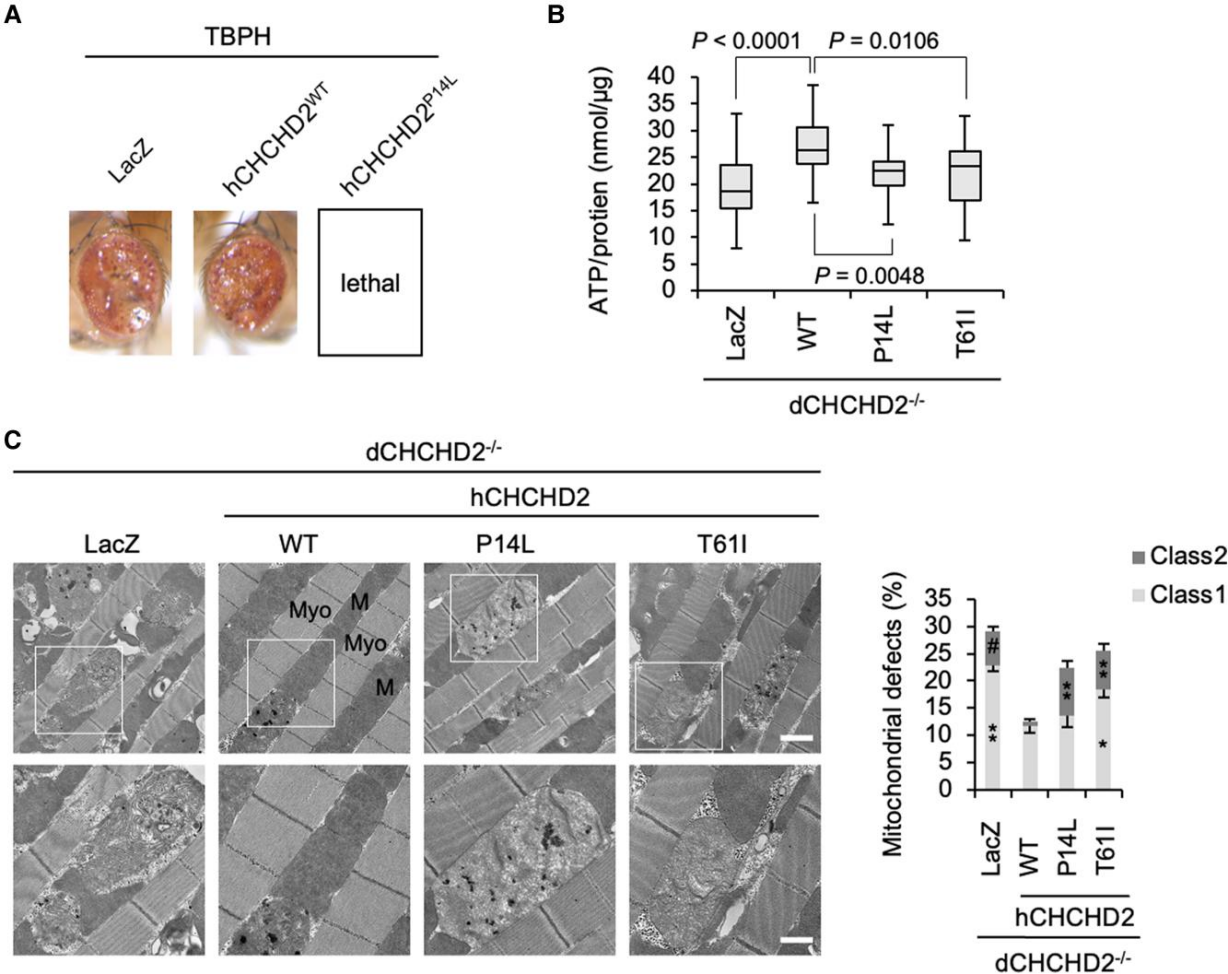
CHCHD2 P14L as a pathogenic mutant in flies. A) CHCHD2 P14L exacerbates neurotoxicity mediated by TBPH overexpression. TBPH was coexpressed with LacZ, hCHCHD2 WT, or P14L in the eyes using the *GMR-GAL4* driver. B) ATP production was not rescued by hCHCHD2 P14L and T61I. The transgenes were driven by *Da-GAL4*. ATP levels normalized with soluble proteins in the thoraces of 30-day-old flies were graphed (mean ± SEM, *n* = 8 independent samples). One-way ANOVA with Tukey–Kramer's test was performed for comparisons among multiple groups. C) TEM images of the indirect flight muscles of 14-day-old adult flies with the indicated genotypes are shown. (Upper) M, mitochondrion; Myo, myofibril. (Lower) Higher-magnification images of the boxed regions in the upper panels. Scale bars, 1 µm (upper) and 500 nm (lower). Mitochondria with abnormal cristae or degenerating mitochondria were quantified using the reported scoring system (7). Mitochondrial defects defined as classes 1 and 2 were counted and presented as percentages (mean ± SEM). *P = 0.039, **P > 0.0001, #*P* > 0.0006 vs. the same class of WT by Tukey–Kramer's test. *n* = 413–551 from 3 to 4 independent samples.

The co-expression of the human CHCHD2 (hCHCHD2) WT did not affect the female eye phenotype induced by TBPH, whereas hCHCHD2 P14L caused lethal damage (Fig. 5A and SI Appendix, Fig. S6B). We previously reported that the loss of dCHCHD2 causes age-dependent mitochondrial degeneration and reduced mitochondrial ATP production in thoracic muscles, which was suppressed by the hCHCHD2 WT but not by T61I associated with PD (7). Therefore, we evaluated the effects of hCHCHD2 P14L. The expression of hCHCHD2 P14L and T61I did not improve ATP production and caused morphological defects in the mitochondria. These findings suggest that hCHCHD2 P14L is a pathogenic variant (Fig. 5B and C).

### Age-dependent mislocalization of CHCHD2 P14L

**Fig. 6.**
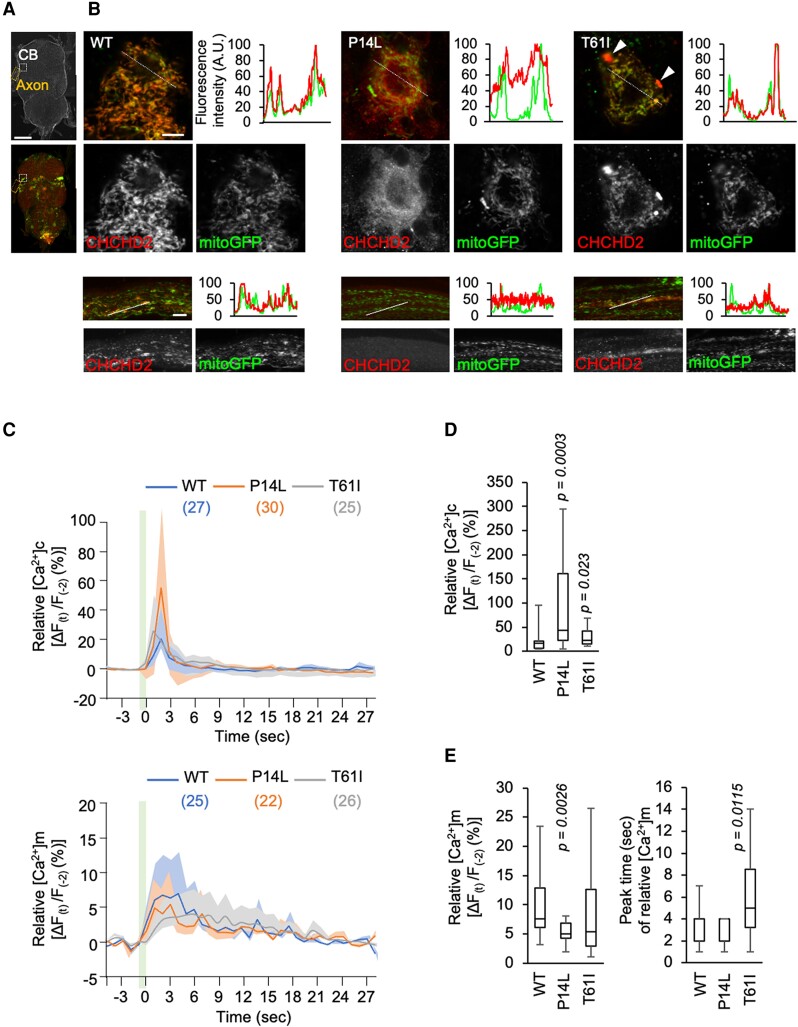
Mitochondrial Ca^2+^-buffering is impaired by CHCHD2 P14L in *Drosophila*. A) Positions of motor neuron cell bodies (CB in boxes) and axons of the ventral nerve cord (VNC, orange boxes) (upper panel, differential interference contrast image). Mitochondria and CHCHD2 were visualized by mitoGFP (green) and anti-CHCHD2 staining (red), respectively (lower). Scale bar, 50 µm. B) Subcellular localization of human CHCHD2 in the VNC motor neuron CBs and axons in 20-day-old flies. Mitochondria and CHCHD2 were visualized by mitoGFP and anti-CHCHD2 staining, respectively. Arrowheads indicate mitoGFP-negative CHCHD2 aggregation. Graphs indicate line profiles of fluorescence intensity along cross-sections in the images. Scale bars, 10 µm. C) Mitochondrial uptake of Ca^2+^ in the PAM DA nerve terminals is impaired by CHCHD2 P14L. Traces (mean ± SEM) of relative fluorescence intensity changes from 2 s before stimulation are shown. A light green bar indicates 40 Hz electrical stimulations (a set of 10 ms intervals at 5 and 15 ms duration). D, E) Graphs indicate relative changes in cytosolic and mitochondrial Ca^2+^ concentration ([Ca^2+^]_c_ and [Ca^2+^]_m_, respectively) at peak time in addition to peak time of [Ca^2+^]_m_. The numbers of flies analyzed are described in parentheses in the graphs. Steel's test vs. WT. GCaMP and mito-GCaMP were driven by *R58E02-GAL4*.

Our previous study suggested that CHCHD2 T61I dissociates from the mitochondria, as observed in a PD case with CHCHD2 T61I and *Drosophila* dopaminergic neurons (9). We evaluated the mitochondrial localization of hCHCHD2 P14L in *Drosophila* dopaminergic neurons. In 5-day-old young adult flies, P14L and T61I signals almost overlapped with the mitochondria-resident GFP reporter mitoGFP signals (SI Appendix, Fig. S10). In 21-day-old flies, both P14L and T61I partially dissociated from the mitochondrial signals (SI Appendix, Fig. S10). Additionally, mitochondrial fragmentation was promoted by P14L and T61I (SI Appendix, Fig. S10A). A similar phenomenon was observed in motor neurons of the ventral nerve cord in 20-day-old flies (Fig. 6A and B). In motor neuron cell bodies and axons, the hCHCHD2 WT was colocalized with mitoGFP (Fig. 6B). In contrast, P14L was localized homogeneously in the cytoplasm and lost clear signals in the axons (Fig. 6B). T61I in the cell body was mostly localized within the mitochondria, but some formed aggregates outside the mitochondria (arrowheads in Fig. 6B). In axons, mitochondrial localization was reduced in T61I compared with the WT (Fig. 6B).

### Mitochondrial Ca^2+^ uptake is impaired by CHCHD2 P14L

We previously observed that the loss of dCHCHD2 affects the mitochondrial Ca^2+^-buffering ability (47). Transient Ca^2+^ influx into neurons was observed in the protocerebral anterior medial (PAM) neuronal terminals projecting to the mushroom bodies upon electrical stimulation. Meanwhile, the mitochondria took up excess Ca^2+^ to moderate cytosolic Ca^2+^ dynamics in the WT (Fig. 6C–E). On the other hand, in the neuronal terminals expressing P14L, Ca^2+^ uptake into the mitochondria was impaired, resulting in a higher cytosolic Ca^2+^ peak (Fig. 6C–E). Neuronal terminals expressing T61I also had a higher cytosolic Ca^2+^ peak than the WT (Fig. 6D); however, the maximum mitochondrial Ca^2+^ uptake was similar to that of the WT (left graph in Fig. 6E). In contrast, mitochondrial Ca^2+^ uptake was delayed in T61I (right graph in Fig. 6E). This change in timing appears to be attributable to the higher cytosolic Ca^2+^ in T61I.

**Fig. 7.**
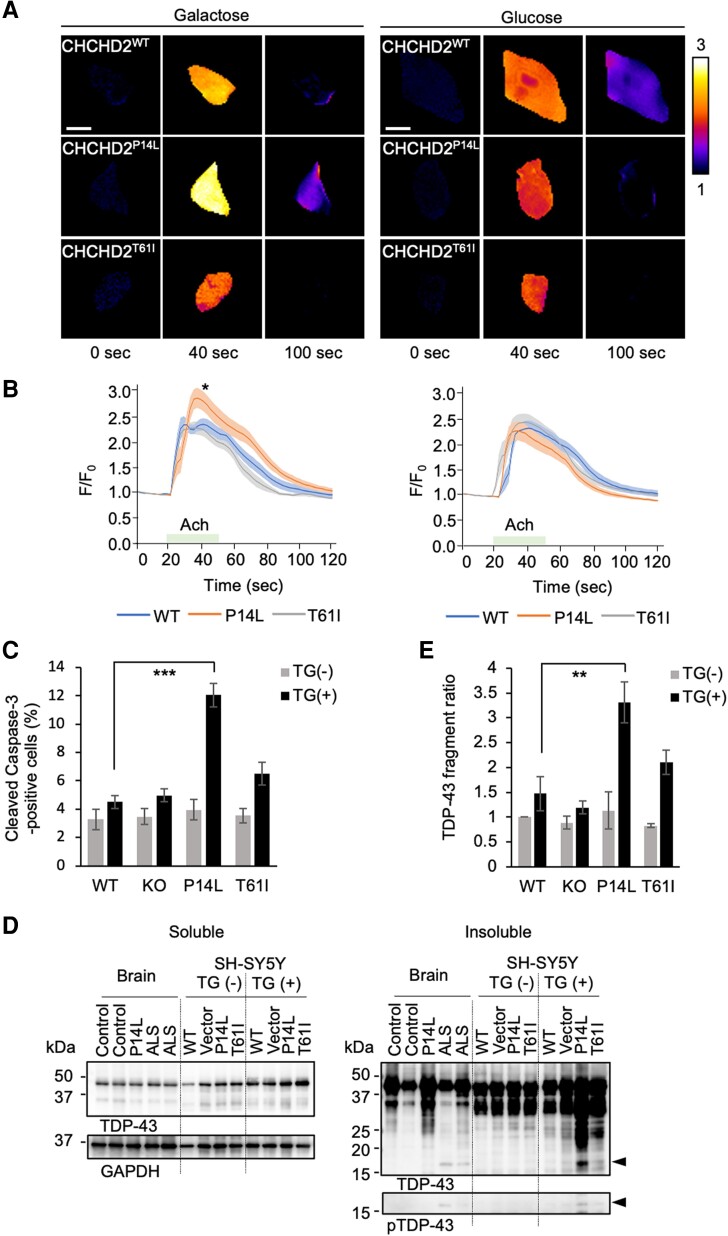
Altered Ca^2+^ dynamics in CHCHD2^−/−^ SH-SY5Y cells expressing CHCHD2 P14L. A, B) Ca^2+^ elevation in the cytoplasm evoked by 10 µM acetylcholine (Ach) in CHCHD2^−/−^ SH-SY5Y cells stably expressing CHCHD2 variants cultured with 5 mM galactose or glucose. Cells were stimulated with Ach for 30 s from 20 s after image acquisition. Color-coded (Δ*F*/*F*_0_, color bar) fluorescence intensity of RCaMP just before stimulation (each left panel), at the time of Ca^2+^ peak (each middle), and at a time of attenuation (each right) are shown. Scale bars, 10 μm. Graphs indicate traces (mean ± SEM) of relative changes in fluorescence intensity from 20 s before Ach stimulation. P14L with galactose showed elevated Ca^2+^ influx when compared to each area under the curve of 20–118 s (*P < 0.040 vs. WT by Welch's *t* test). C) Elevated cytosolic Ca^2+^ by CHCHD2 P14L promotes cell death. Cells treated with 100 nM thapsigargin (TG) for 24 h in a galactose-containing medium were immunostained with anti-cleaved caspase-3. The percentage (mean ± SEM) of cleaved caspase-3-positive cells is graphed (***P < 0.0001 by Dunnett's test. More than 1,350 cells from three biological replicates). Representative staining results are shown in Fig. S13A. D) Elevated cytosolic Ca^2+^ by CHCHD2 P14L promotes cleavage and insolubilization of TDP-43. CHCHD2^−/−^ SH-SY5Y cells stably expressing CHCHD2 variants or mock vector were treated with 100 nM thapsigargin (TG+) or DMSO (TG−) as in (C). Cell lysate along with human autopsied brain tissues (control, ALS with CHCHD2 P14L, and sporadic ALS) fractionated with a lysis buffer containing 1% Triton X-100 was analyzed by western blotting with the indicated antibodies. Arrows indicate a ∼17-kDa insoluble TDP-43 C-terminal fragment commonly detected in P14L and sporadic ALS, which was phospho-Ser409/410-positive. E) Increased production of the TDP-43 C-terminal fragment of approximately 17 kDa in cells expressing CHCHD2 P14L. The relative band intensity (mean ± SEM, *n* = 3 biological replicates) of the TDP-43 C-terminal fragment detected by anti-TDP-43 (arrowhead) in D) was graphed after normalization with GAPDH in the soluble fraction. **P < 0.0066 by Dunnett's test.

Next, we analyzed whether the altered Ca^2+^ dynamics in *Drosophila* can be observed in human cells. In SH-SY5Y cells, glycolytic respiration was dominant in media containing glucose, whereas mitochondrial respiration was dominant in media containing galactose (48). Under the condition in which *CHCHD2^−/−^* SH-SY5Y cells expressing each CHCHD2 variant were cultured in a galactose-containing medium, cytosolic Ca^2+^ elevation was monitored after stimulation with acetylcholine (Fig. 7A and B) (49). Cytoplasmic Ca^2+^ was elevated to higher levels in cells expressing CHCHD2 P14L than in the other cells (left graph in Fig. 7B). The higher elevation of cytosolic Ca^2+^ in cells expressing CHCHD2 P14L disappeared under culture condition in a glucose-containing medium (right graph in Fig. 7B). These results suggest that CHCHD2 P14L affects the mitochondrial Ca^2+^-buffering function when mitochondrial respiratory activity is dominant.

Mitochondrial Ca^2+^ uptake involves the mitochondrial calcium uniporter (MCU) and its regulator MCUR (50). We tested the possibility that CHCHD2 directly binds to them. However, MCU and MCUR did not co-precipitate with CHCHD2 by immunoprecipitation (SI Appendix, Fig. S11A). Similar results were obtained by immunoprecipitation after chemical cross-linking.

### Elevated cytoplasmic Ca^2+^ promotes aggregation of TDP-43

TDP-43 processing has been reported to involve Ca^2+^-activated calpain and apoptotic caspases (caspase-3/6/7/8) in ALS (51–53). We treated *CHCHD2^−/−^* SH-SY5Y cells expressing each variant of CHCHD2 with the sarcoplasmic/ER Ca^2+^ ATPase inhibitor thapsigargin, which induces ER stress and increases the cytosolic Ca^2+^ concentration. In cells expressing P14L, thapsigargin treatment promoted the release of cyt C and CHCHD2 (SI Appendix, Fig. S11B and C) and activated caspase-3 (Fig. 7C and SI Appendix, Fig. S12A). Under this condition, the 1% Triton X-100-insoluble TDP-43 fragments increased in cells expressing P14L, including the C-terminal fragment of approximately 17 kDa, as observed in the brain tissues of patients with sporadic ALS (arrowheads in Fig. 7D and E). Moreover, an increase in the cytoplasmic aggregation of TDP-43 was observed in cells expressing P14L compared with the WT (Fig. S12B). The appearance of the 17-kDa insoluble C-terminal fragment was dependent on both calpain and caspase activities (SI Appendix, Fig. S12C).

## Discussion

Given that *CHCHD10*, a paralog of *CHCHD2*, is the causative gene for ALS-FTLD, we hypothesized that CHCHD2 mutations may contribute to the pathogenesis of ALS. Based on this hypothesis, we screened a Japanese cohort of patients with ALS for *CHCHD2* variants. We identified two previously unreported variants: −8T > G and c.41C > T (p.P14L). Pathological analysis revealed accumulation of TDP-43 aggregates in neurons and glial cells, which is a typical pathology of ALS. Since genomic information on the families of patients with these variants is not available, we cannot determine whether de novo mutations directly related to the development of ALS have occurred or whether these mutations increase the risk of ALS. The patient with the −8T > G variant was found to have another variant in the *SETX* gene. The allele frequency of the −8T > G variant in the JaCALS cohort showed no difference compared to that in the general East Asian population, and there were no differences observed in mRNA and protein levels compared to healthy individuals, suggesting a low pathogenic potential. Conversely, the c.41C > T variant showed a mild decrease in mRNA and protein levels and a significant change in protein localization, leading to suspicion of its pathogenicity. c.41C > T creates a missense mutation, P14L, in the N-terminal region of CHCHD2, which could have an effect on the protein, similar to ALS-associated CHCHD10 P12S (8). In this experiment, the effect of the P14L variant on the function of CHCHD2 was analyzed in detail in human cells and *Drosophila*, compared to T61I associated with PD.

CHCHD2 P14L and T61I, unlike the CHCHD2 WT, failed to restore mitochondrial function and suppress mitochondrial degeneration caused by the loss of *CHCHD2* and *CHCHD10* ortholog CG5010 (dCHCHD2) in *Drosophila*. These observations strongly suggested that P14L is pathogenic, similar to T61I. Furthermore, human CHCHD2 P14L and TBPH showed strong genetic interactions in *Drosophila*. In particular, the loss of dCHCHD2 also enhanced cytotoxicity caused by the overexpression of TBPH or human TDP-43, and further promoted the translocation of overexpressed TBPH to the cytoplasm. Mitochondria lacking dCHCHD2 produce excessive ROS, which may cause the cytoplasmic translocation and aggregation of TBPH owing to oxidative stress (7). In the absence of dCHCHD2, endogenous TBPH aggregated in the nucleus and near and inside the mitochondria in thoracic muscle tissues. These TBPH aggregates were in the vicinity of polyUb-positive aggregates but did not merge. No significant accumulation of 1% sarkosyl-insoluble TBPH was observed in the absence of dCHCHD2. Skein-like inclusions in ALS have been suggested to consist of a mixture of histone deacetylase 6/microtubule-dependent aggresomes and aggregations induced by LLPS (16). The aggregation of TBPH observed in flies, which is polyUb-negative and 1% sarkosyl-soluble, may occur via LLPS.

CHCHD2 P14L has a reduced ability to form complexes with CHCHD10 and is prone to leakage from the mitochondria into the cytoplasm. Cytoplasmic mislocalization increases with stress in cultured cells and with ageing in *Drosophila*. Furthermore, cells expressing CHCHD2 P14L showed higher cytosolic Ca^2+^ concentrations upon stimulation, which appeared to be due to a reduced mitochondrial Ca^2+^-buffering capacity. A similar phenomenon has been observed in *dCHCHD2*-knockout flies (47), suggesting that P14L is a loss-of-function mutation. However, TDP-43 fragmentation and caspase-3 activation after thapsigargin treatment were prominent in cells expressing CHCHD2 P14L but not in *CHCHD2* knockout cells. This observation suggests that P14L may also possess the properties of a gain-of-function mutation. Since CHCHD2 can bind to cyt C, P14L may actively translocate cyt C into the cytoplasm and promote the activation of apoptosis-related caspase-3/-7 (7). Furthermore, elevated cytosolic Ca^2+^ levels may activate calpain, which, together with caspases, can promote TDP-43 processing and facilitate the accumulation of TDP-43 (52). In the present study, we detected a 17-kDa fragment of the TDP-43 C-terminal in the brain tissues of patients with ALS and SH-SY5Y cells treated with thapsigargin. This fragment was generated in a manner dependent on calpain and caspase activity. The protein sequence of pS409/410-positive TDP-43 fragments in the sarkosyl-insoluble fraction of FTD brain tissue revealed C-terminal fragments of TDP-43 with D219 and D247 at the N-terminus (54). These fragments with N-terminal D219 and D247, detected in cultured cells as approximately 18 and 17 kDa proteins, respectively, were highly insoluble and trapped endogenous TDP-43, interfering with its splicing function (54, 55). In contrast, calpain cleaves TDP-43 between L243 and C244, creating a 17-kDa C-terminal fragment (52). These 17-kDa C-terminal TDP-43 fragments may contribute to TDP-43 aggregation and ALS pathogenesis (55). On the other hand, the possibility that ALS pathogenesis arises from haploinsufficiency cannot be ruled out. Heterozygous rare variants within the predicted mitochondrial targeting sequence of CHCHD2 are linked to PD and Lewy body disease (56). Some of these variants may involve amino acid substitutions that prevent proper mitochondrial localization, suggesting that CHCHD2 haploinsufficiency could contribute to proteostasis disruption (57).

The protein expression of CHCHD2 P14L and T61I was reduced in the sarkosyl-soluble fraction of the human brain, suggesting their potential as loss-of-function mutations. P14L is likely subject to active degradation due to its mislocalization in the cytosol, whereas T61I shows reduced expression due to insolubilization within the mitochondria. Interestingly, following arsenite treatment, the CHCHD2 P14L signal intensified in the nucleus, suggesting its role in the nucleus (58). Further analysis is required to explore this possibility. Very recently, decreased CHCHD2 expression and the presence of cytosolic TDP-43 aggregates have been reported in astrocytes derived from ALS/Parkinsonism-Dementia Complex (ALS/PDC) iPS cells (59). ALS/PDC is a rare and complex neurological disorder observed in the western Pacific islands, including Japan, Guam, and Papua. Its symptoms are similar to those seen in ALS and PD, with inclusions of α-synuclein and TDP-43. The TDP-43 aggregation mechanism we identified in this study supports this finding.

**Fig. 8.**
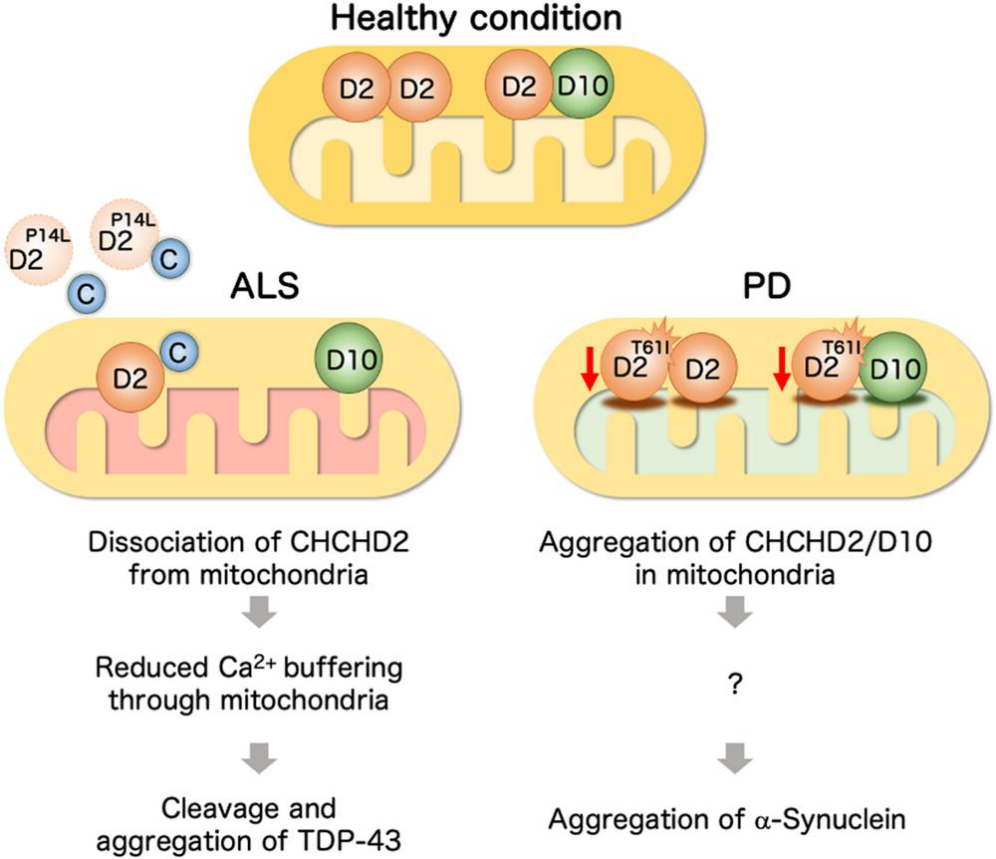
Working hypothesis. (Left) During mitochondrial stress, ALS-associated P14L mislocalizes CHCHD2 to the cytoplasm, facilitating the release of cyt C (C) from the mitochondria and subsequent caspase activation. Reduced mitochondrial Ca^2+^-buffering function in the absence of mitochondrial CHCHD2 promotes the elevation of cytoplasmic Ca^2+^, leading to TDP-43 fragmentation and aggregation. (Right) In PD, aggregate-prone T61I insolubilizes with CHCHD10 in the mitochondria. The aggregation of CHCHD2/D10 causes an integrated stress response. A chronic stress response may cause α-synuclein aggregation.

In summary, in this study, we found differences in protein properties between T61I causing PD and P14L found in ALS. T61I tends to insolubilize within mitochondria, probably involving CHCHD10 because of the higher binding of T61I to CHCHD10. The observation that both CHCHD2 and CHCHD10 were insoluble in autopsied brains supports this hypothesis. Stress from such insolubilized proteins in the mitochondria could be transmitted to the nucleus and alter proteostasis in cells (60, 61). Presumably, chronic unfolded protein stress alters the intracellular environment in such a way as to increase the risk of α-synuclein aggregation (62) (Fig. 8). In contrast, P14L is more likely to leak into the cytoplasm and affect Ca^2+^ uptake into the mitochondria. Elevated cytosolic Ca^2+^ is known to cause hyperexcitation of motor neurons and subsequent neuronal cell death (63). Elevated cytosolic Ca^2+^ levels promote calpain activation and TDP-43 processing (52). Increased cell death signaling may also promote caspase activation and further processing of TDP-43, leading to aggregation (51) (Fig. 8). The mitochondrial Ca^2+^ uniporter MCU and its regulator MCUR were not physically bound to CHCHD2. Elucidating the mechanism of mitochondrial Ca^2+^ regulation by CHCHD2 is a future challenge, and a potential new drug discovery target for the treatment of ALS.

## Materials and methods

### Human genetic analysis

Genetic analysis of 944 ALS patients enrolled in the Japanese ALS registry JaCALS by December 2014 was performed using the methods presented in a previous study (41). These patients were of Japanese ancestry and were diagnosed with definite, probable, probable laboratory-supported, or possible ALS using the revised El Escorial criteria. The research plan was reviewed and approved by the Ethics Committee, Aichi Medical University School of Medicine (approval number: 2021-083), and permission to conduct the study was granted at all participating institutions based on this review and approval. Targeted resequencing was performed in the following 28 genes related to ALS: *SOD1, ALS2, SETX, SPG11, FUS, VAPB, ANG, TARDBP, FIG4, OPTN, VCP, UBQLN2, SIGMAR1, DAO, NEFH, DCTN1, TAF15, EWSR1, PRPH, GRN, CHMP2B, ZNF512B, PFN1, ATXN2, TFG, C9orf72, RNF19A,* and *SQSTM1*. *CHCHD2* was sequenced separately using the Sanger method. For the patient with P14L, no mutations were found in 28 genes related to ALS. The patient with −8T > G carried a variant (c.628A > G, p.I210 V) in *SETX*, although its pathogenicity is not known. The GGGGGCC repeat expansion of C9orf72 was not detected in either case.

### Human brain tissues

The study included five patients with ALS and three controls without neurodegenerative diseases. Age, sex, disease duration, postmortem interval, and the availability of frozen brain tissue are shown in Table S2. The diagnosis was confirmed by neuropathological examination (64). This study was approved by the Ethics Committee of the Juntendo University School of Medicine (approval number: M19-0235). Informed consent was obtained from donors or authorized representatives for the use of tissues in this study.

### Plasmids and antibodies

Human CHCHD2 P14L was generated using the QuikChange site-directed mutagenesis kit (Agilent Technologies, Santa Clara, CA, USA) and cloned into pMXs-puro for SH-SY5Y cells or pUASTattB for *Drosophila* transgenics. Antibodies against TBPH were raised in rabbits using recombinant TBPH protein purified from bacteria BL21 harboring pREST-dTDP (307-531 aa) (46) and affinity purified with the antigen. All antibodies used in this study are listed in Table S3.

### Drosophila genetics

Fly culture and crosses were performed on standard fly food containing yeast, cornmeal, and molasses. Flies were raised at 25°C unless otherwise stated. Transgenic lines carrying hCHCHD2 P14L were generated on the *w*^1118^ background using the PhiC31 system and the Gateway System (BestGene). All other fly stocks and *GAL4* lines used in this study, including *dCHCHD2 null* (7)*, hCHCHD2 WT* (7)*, hCHCHD2 T61I* (7), *TBPH^G2^* (65), *UAS-TBPH-FLAG* (66), *UAS-TDP-43-FLAG* (66), *PINK1^B9^* (67), *R58E02-GAL4* (68), and *UAS-mito-GCaMP6* (47) were obtained from the Bloomington *Drosophila* Stock Center and have previously been described. The detailed genotypes used in this study are listed in SI Appendix.

### Establishment of CHCHD2^−/−^ SH-SY5Y cell lines expressing compensatory hCHCHD2

Exon 3 of *CHCHD2* in SH-SY5Y cells was disrupted by genome editing. The detailed procedures for genome editing are described in SI Appendix. *CHCHD2^−/−^* SH-SY5Y cells were retrovirally transfected with pMXs-puro harboring hCHCHD2 WT, P14L, T61I, or LacZ as a mock vector. Transfected cells were then selected with 1 µg/mL puromycin for 48 h. Single cell lines were obtained by limited dilution, and clones stably expressing endogenous CHCHD2 were selected by western blotting.

### Biochemical fractionation

Sequential biochemical fractionation of autopsy brain tissues and cultured cells was performed as previously described (9). The detailed procedures are described in SI Appendix.

### *Drosophila* experiments

The detailed procedures for the climbing assay, ATP measurement, whole-mount immunostaining, histochemistry, transmission electron microscopy, and immunoelectron microscopy are described in SI Appendix.

### Ca^2+^ imaging of *Drosophila* DA neurons

*Drosophila* Ca^2+^ imaging was performed as previously described (47). Briefly, each fly head was held in a hole (1-mm in diameter) on a plastic plate (Cat. No. 12-547, Fisherbrand, Thermo Fisher Scientific, Waltham, MA, USA) using nail polish. Subsequently, the mouth parts and cuticles around the antennae were removed using tweezers. Ca^2+^ imaging of brain DA neurons expressing mito-GCaMP6 or GCaMP6f was performed in Ca^2+^-free HL-3 using an Eclipse FN1 microscope (Nikon, Tokyo, Japan) equipped with an electrical stimulation setup containing SEN-3401 (Nihon Kohden, Tokyo, Japan) and SS-104J (Nihon Kohden). The lateral side of the antennal lobe was stimulated using a glass electrode (cat. No. W3 64-0792, Warner Instruments, Hamden, CT, USA) with a 100-µm tip diameter made by an electrode puller (P-97, Sutter Instrument, Novato, CA, USA). GCaMP images were recorded for 1 min (1 frame/0.03 s), and 40 Hz electrical stimulation (5 V with 15-ms duration 10-ms intervals) was applied for 1 s in the antennal regions. Each imaging session was performed within 10 min after dissection. Ca^2+^ imaging data were processed using NIS-Elements software (Nikon, ver. AR-4.40.00), ImageJ-Fiji (ver. 1.0), and Excel (ver. 2010; Microsoft, Redmond, WA, USA).

### Imaging of SH-SY5Y cells

For Ca^2+^ imaging of *CHCHD2^−/−^* SH-SY5Y cells expressing each CHCHD2 variant, the cells were cultured in glass-bottom dishes (Mattek, Ashland, MA, USA) and transfected with pCAG cyto-RCaMP1h (Addgene number: 105014). Cells were perfused with HEPES-buffered Krebs–Ringer solution using a peristaltic pump at room temperature and imaged using an Eclipse TE2000-E inverted microscope (Nikon) equipped with a xenon lamp (Hamamatsu Photonics, Hamamatsu, Japan) and an sCMOS camera (Zyla 5.5, Andor, Belfast, UK). To evoke cytosolic Ca^2+^ elevation, 10 µM acetylcholine was administered via perfusion. Ca^2+^ imaging data were acquired using MetaMorph software (ver. 7.10.2.240; Molecular Devices, San Jose, CA, USA) and processed using ImageJ-Fiji (ver. 2.14.0/1.54f).

Detailed procedures for cell imaging of CHCHD2, along with cyt C release and counting, are described in SI Appendix.

### Statistics and reproducibility

Error bars in bar graphs represent the mean ± standard error of the mean (SEM). Box-and-whisker plots indicate the 25th–75th percentiles; horizontal lines in the boxes indicate the 50th percentile, and whiskers represent the maximum and minimum values. A two-tailed Student's *t* test or one-way repeated-measures analysis of variance (ANOVA) was used to determine significant differences between two groups or among multiple groups, respectively, unless otherwise indicated. If a significant result was determined using ANOVA (*P* < 0.05), the mean values of the control and specific test groups were analyzed using the Tukey–Kramer's test. The data distribution was assumed to be normal; however, this was not formally tested. Abnormal mitochondria (Fig. 5C) were counted and blindly classified by H.M. and Jun-Yi Liu. Blinding was not performed in the other experiments.

## Supplementary Material

pgae319_Supplementary_Data

## Data Availability

All relevant data are provided in the manuscript and its Supplementary material.
