# Supplementary material for: CHCHD2 P14L, found in amyotrophic lateral sclerosis, exhibits cytoplasmic mislocalization and alters Ca2+ homeostasis
Source: PNAS Nexus. 2024 Jul 30;3(8):pgae319. doi: 10.1093/pnasnexus/pgae319 (PMC11316225; doi:10.1093/pnasnexus/pgae319)
Supplement: pgae319_Supplementary_Data [file pgae319_supplementary_data.pdf]

PNAS nexus

Supporting Information for

CHCHD2 P14L, found in amyotrophic lateral sclerosis, exhibits cytoplasmic mislocalization and alters Ca^{2+} homeostasis

Aya Ikeda, Hongrui Meng, Daisuke Taniguchi, Muneyo Mio, Manabu Funayama, Kenya Nishioka, Mari Yoshida, Yuanzhe Li, Hiroyo Yoshino, Tsuyoshi Inoshita, Kahori Shiba-Fukushima, Yohei Okubo, Takashi Sakurai, Taku Amo, Ikuko Aiba, Yufuko Saito, Yuko Saito, Shigeo Murayama, Naoki Atsuta, Ryoichi Nakamura, Genki Tohnai, Yuishin Izumi, Mitsuya Morita, Asako Tamura, Osamu Kano, Masaya Oda, Satoshi Kuwabara, Toru Yamashita, Jun Sone, Ryuji Kaji, Gen Sobue, Yuzuru Imai, Nobutaka Hattori

Yuzuru Imai
E-mail: yzimai@juntendo.ac.jp

Nobutaka Hattori
E-mail: nhattori@juntendo.ac.jp

This PDF file includes:

- Supporting text
- Figures S1 to S12
- Tables S1 to S3
- SI References

Supporting Information Text

SI Materials and Methods

Cell reagents

Thapsigargin (33637-31) and z-VAD-fmk (3188-v) were purchased from Nacalai Tesque (Kyoto, Japan) and the Peptide Institute (Ibaraki, Japan), respectively. Calpain inhibitor I (no. 14921) and caspase-3/7 inhibitor I (no. 14464) were purchased from Cayman Chemical Co. (Ann Arbor, MI, USA).

Generation of CHCHD2$^{-/-}$ SH-SY5Y cell lines

Exon 3 of *CHCHD2* in SH-SY5Y cells was disrupted by genome editing. The GeneArt CRISPR Search and Design Tool (https://www.thermofisher.com/crisprdesign) and CRISPRdirect (https://crispr.dbcls.jp) (1) were used to design the sgRNAs. Oligonucleotides with *Bpi*I linkers (5'-caccGAAACTGTTTGATCTCATAG and 5'-aaacCTATGAGATCAAACAGTTTC) were annealed and ligated into pSpCas9(BB)-2A-Puro (PX459) (Addgene #62988) (2). SH-SY5Y cells transfected with PX459 by electroporation using NEPA21 (Nepagene, Ichikawa, Japan) were treated with 2 µg/ml puromycin for 48 h after transfection. Single-cell lines were obtained by limited dilution, and gene disruption was confirmed by Sanger sequencing and western blotting using an anti-CHCHD2 antibody.

Biochemical fractionation

Sequential biochemical fractionation of autopsy brain tissues and cultured cells was performed using Buffer A (10 mM Tris–HCl, pH 7.4, 0.8 M NaCl, 1 mM EGTA, 10% sucrose) supplemented with a complete protease inhibitor cocktail, as previously described (3). The 1% sarkosyl-insoluble and 1% Triton X-100-insoluble pellets were analyzed as insoluble proteins in the brain tissues and cultured cells, respectively. To fractionate the cytoplasm, mitochondria, and nuclei of *CHCHD2*$^{-/-}$ SH-SY5Y cells with complementary expression of hCHCHC2, the cells (6.6×10^6 cells) were fractionated using a cell fractionation kit (ab109719, Abcam, Cambridge, UK).

RT–PCR

Total RNA extracted from the frontal lobes of the brain was reverse transcribed using random 9-mer primers and M-MLV reverse transcriptase (M1701, Promega, Madison, WI, USA). Subsequent SYBR green-based real-time PCR using a KAPA SYBR Fast kit (KAPA Biosystems, Roche Diagnostics, Rotkreuz, Switzerland) was performed using a QuantStudio 7 Flex system (Thermo Fisher Scientific). The primer pairs used for *CHCHD2* were as follows: forward, 5'-GATGCCGCGTGGAAGCCGAA; reverse, 5'-TGGTGGCTGAGCGACTGGTG (3). For *CHCHD10*, the primer pairs were as follows: forward, 5'-AGAGTGACCTGTCCCTGTGTGA; reverse, 5'-GACCATGGTAGTACTTGCACTGCTT. CHCHD2 transcript levels were normalized to those of corresponding *ACTB* transcripts (4).

Drosophila climbing assay

Vials (25 mm diameter × 180 mm height) containing 20–25 flies were gently tapped onto the table and left standing for 18 s. The number of flies climbing at least 60 mm was recorded.

ATP measurement in *Drosophila*

Three male fly heads were homogenized in 30 µl of homogenization buffer (6 M guanidine, 100 mM Tris-HCl, pH 7.8, and 4 mM EDTA) using a motor-driven pestle. After centrifugation at 20,000 × g for 10 min, the supernatants were diluted 1:100 and 1:5 with distilled water for ATP and protein quantification, respectively. The ATP content and protein concentration were measured using a CellTiter-Glo luminescent cell viability assay kit (G7570, Promega) and a protein assay bicinchoninate (BCA) kit (06385-00, Nacalai Tesque), respectively.

Whole-mount immunostaining, histochemistry, transmission electron microscopy (TEM) analysis and immunoelectron microscopy for *Drosophila*

For whole-mount immunostaining, brain tissues or ventral ganglia fixed with 4% paraformaldehyde/PBS solution were washed three times with PBS containing 0.3% Triton X-100 (PBS/Tx), blocked with 1% normal

goat serum, and stained with the indicated antibodies overnight at 4 °C. After washing with PBS/Tx three times and subsequently incubating with secondary antibodies conjugated to the fluorescent dye, the tissues were mounted on glass slides using the Fluoro-KEEPER antifade reagent (12745-74, Nacalai Tesque). The dissected thoraxes were fixed with 4% paraformaldehyde/PBS, washed three times, and embedded in paraffin. Immunohistochemical staining was performed on 4-μm-thick muscle tissue sections using the indicated primary antibodies. For anti-TBPH staining of the paraffin sections, a Target Retrieval Solution (pH 9.0, S2367, Dako, Agilent Technologies, Santa Clara, CA, USA) and Can Get Signal Immunoreaction Enhancer Solution A (NKB-501, Toyobo, Osaka, Japan) were used to enhance the immunosignals. Images were acquired using laser-scanning microscope systems: TCS-SP5 (Leica Microsystems, Wetzlar, Germany) and LSM880 with Airyscan (Carl Zeiss, Oberkochen, Germany). The aggregates of TBPH and polyUb were thresholded and binarized using ImageJ software. The aggregates per unit area were quantified using a particle analyzer tool. Pearson correlation coefficients between CHCHD2 and mitoGFP signals were analyzed with the ImageJ plugin Colocalization Finder. A similar analysis was also performed with the ImageJ Coloc 2 tool on human tissue sections and cultured cells. The TEM images were obtained using an electron microscope (HT7700; Hitachi, Tokyo, Japan) at the Laboratory of Ultrastructural Research, Juntendo University.

Immunoelectron microscopy (IEM) was performed using a Tokai electron microscope. Briefly, *Drosophila* thoracis muscle sections on gold disks frozen in liquid propane at -175 °C were substituted with 0.2% glutaraldehyde in ethanol and 3% distilled water at -80 °C overnight, -20 °C for 2 h, and 4 °C for 1 h. Samples were dehydrated with 100% ethanol and infiltrated with resin. Approximately 90-nm-thick sections cut with a cryo-ultramicrotome were incubated with rabbit anti-TBPH (1:50) at RT for 2 h and then with goat anti-rabbit IgG conjugated with 10-nm colloidal gold particles (1:100) at RT for 1 h. After washing with PBS, the samples were placed in 2% glutaraldehyde in 0.1 M phosphate buffer, stained with 2% uranyl acetate, and secondary-stained with a lead stain solution. IEM images were obtained using an electron microscope (JEM-1400Plus, JEOL, Akishima, Japan).

Mitochondrial import assay

The mitochondrial import assay was performed as previously described, with slight modifications (5). Briefly, CHCHD2 was synthesized using the TnT® Quick Coupled Transcription/Translation System (L1170, Promega) on a 50 μl reaction scale, and the same procedure was performed with the pcDNA3.1 empty vector as a mock control. Mitochondria were isolated from $CHCHD2^{-/-}$ SH-SY5Y cells (4×10^7 cells). The cells were trypsinized, washed with PBS, suspended in 4 ml of M buffer (220 mM mannitol, 70 mM sucrose, 5 mM HEPES-KOH, pH 7.4, 1 mM EGTA-KOH) supplemented with a protease inhibitor cocktail (03969-21, Nacalai Tesque), and homogenized in Dounce tissue grinders with a tight pestle for 60 strokes. The cell disruption solution was centrifuged at 600 × g for 5 min at 4 °C to remove nuclei, and the resulting supernatant was collected in a new tube. After further centrifugation at 600 × g for 5 min at 4 °C, the supernatant containing the mitochondria was collected in 1.5 ml tubes. The supernatant was centrifuged at 8,000 × g for 10 min at 4 °C to precipitate mitochondria. The mitochondrial pellet was suspended in 500 μl of M buffer, washed with an additional 6,000 g centrifugation for 10 min at 4 °C, and resuspended in 400 μl of M buffer. After measuring the protein concentration using a BCA protein assay kit (06385-00, Nacalai Tesque), 20 μg of mitochondria were centrifuged at 8,000 × g for 5 min at 4 °C. Five microliters of the TNT reaction mixture containing synthesized CHCHD2 was added to 20 μg of pelleted mitochondria, incubated at 600 rpm at 30 °C, and sampled at 1-, 10-, and 30-min time points, with 5 μl taken at each interval. In parallel, mitochondria preincubated with 200 μM CCCP for 5 min to decrease the mitochondrial membrane potential ($\Delta\Psi m$) were also prepared to confirm $\Delta\Psi m$-dependent CHCHD2 import. Mitochondria were treated with proteinase K (20 μg/ml) for 20 min on ice to remove CHCHD2 outside the mitochondria. Triton X-100 (0.5%) was added simultaneously with proteinase K treatment to digest CHCHD2 inside the mitochondria. CHCHD2 and Tim23 were detected using specific antibodies.

Cell imaging and counting

For cyt C and CHCHD2 release assays, Z-stacked images acquired using a Keyence BZ-X800 fluorescence microscope (Osaka, Japan) were thresholded and binarized using ImageJ software. Cell boundaries were delineated using the ImageJ Watershed tool, and the number of cells was determined by DAPI staining. Cells with signals of cyt C or CHCHD2 measuring ≥ 7.3 μm^2 were defined as mitochondrial release-positive cells. The number of cells with TDP-43 aggregation was visually measured by taking three

random views using a Keyence BZ-X800 fluorescence microscope after staining with the anti-TDP-43 antibody and DAPI.

***Drosophila* genotypes used in this study**

Fig. 4A
Male
w^{1118}/Y; GMR-GAL4/UAS-LacZ (LacZ, Control)
w^{1118}, CG5010^{null}/Y; GMR-GAL4/UAS-LacZ (dCHCHD2$^{-/-}$, LacZ, Control)
w^{1118}, PINK1^{B9}/Y; GMR-GAL4/UAS-LacZ (dPINK1$^{-/-}$, LacZ, Control)
w^{1118}/Y; GMR-GAL4/UAS-TBPH-FLAG (TBPH, Control)
w^{1118}, CG5010^{null}/Y; GMR-GAL4/UAS-TBPH-FLAG (dCHCHD2$^{-/-}$, TBPH, Control)
w^{1118}, PINK1^{B9}/Y; GMR-GAL4/UAS-TBPH-FLAG (dPINK1$^{-/-}$, TBPH, Control)

Female
w^{1118}/w^{1118}; GMR-GAL4/UAS-LacZ (LacZ, Control)
w^{1118}, CG5010^{null}/w^{1118}, CG5010^{null}; GMR-GAL4/UAS-LacZ (dCHCHD2$^{-/-}$, LacZ, Control)
w^{1118}, PINK1^{B9}/w^{1118}, PINK1^{B9}; GMR-GAL4/UAS-LacZ (dPINK1$^{-/-}$, LacZ, Control)
w^{1118}/w^{1118}; GMR-GAL4/UAS-TBPH-FLAG (TBPH, Control)
w^{1118}, CG5010^{null}/w^{1118}, CG5010^{null}; GMR-GAL4/UAS-TBPH-FLAG (dCHCHD2$^{-/-}$, TBPH, Control)
w^{1118}, PINK1^{B9}/w^{1118}, PINK1^{B9}; GMR-GAL4/UAS-TBPH-FLAG (dPINK1$^{-/-}$, TBPH, Control)

Fig. 4B, Fig. S6A
w^{1118}/Y; UAS-mitoGFP/UAS-LacZ; MHC-GAL4/+ (dCHCHD2$^{+/+}$, LacZ)
w^{1118}, CG5010^{null}/Y; UAS-mitoGFP/UAS-LacZ; MHC-GAL4/+ (dCHCHD2$^{-/-}$, LacZ)
w^{1118}/Y; UAS-mitoGFP/+; MHC-GAL4/UAS-TDP-43-FLAG (dCHCHD2$^{+/+}$, TDP-43)
w^{1118}, CG5010^{null}/Y; UAS-mitoGFP/+; MHC-GAL4/UAS-TDP-43-FLAG (dCHCHD2$^{-/-}$, TDP-43)

Fig. 4C-E
w^{1118}/Y; UAS-mitoGFP/UAS-LacZ; D42-GAL4/+ (dCHCHD2$^{+/+}$, LacZ)
w^{1118}, CG5010^{null}/Y; UAS-mitoGFP/UAS-LacZ; D42-GAL4/+ (dCHCHD2$^{-/-}$, LacZ)
w^{1118}/Y; UAS-mitoGFP/UAS-TBPH-FLAG; D42-GAL4/+ (dCHCHD2$^{+/+}$, TBPH)
w^{1118}, CG5010^{null}/Y; UAS-mitoGFP/UAS-TBPH-FLAG; D42-GAL4/+ (dCHCHD2$^{-/-}$, TBPH)
w^{1118}/Y; UAS-mitoGFP/+; D42-GAL4/UAS-TDP-43-FLAG (dCHCHD2$^{+/+}$, TDP-43)
w^{1118}, CG5010^{null}/Y; UAS-mitoGFP/UAS-TBPH-FLAG; D42-GAL4/UAS-TDP-43-FLAG (dCHCHD2$^{-/-}$, TDP-43)

Fig. 5A
w^{1118}/Y; GMR-GAL4/UAS-TBPH-FLAG; UAS-LacZ/+ (LacZ, TBPH)
w^{1118}, CG5010^{null}/Y; GMR-GAL4/UAS-TBPH-FLAG; UAS-CHCHD2 WT/+ (hCHCHD2^{WT}, TBPH)
w^{1118}, CG5010^{null}/Y; GMR-GAL4/UAS-TBPH-FLAG; UAS-CHCHD2 P14L/+ (hCHCHD2^{P14L}, TBPH)

Fig. 5B, C, Fig. S6B
w^{1118}, CG5010^{null}/Y; +/UAS-LacZ; MHC-GAL4/+ (LacZ)
w^{1118}, CG5010^{null}/Y; +/+; MHC-GAL4/UAS-CHCHD2 WT/+ (hCHCHD2^{WT})
w^{1118}, CG5010^{null}/Y; +/+; MHC-GAL4/UAS-CHCHD2 P14L/+ (hCHCHD2^{P14L})
w^{1118}, CG5010^{null}/Y; +/+; MHC-GAL4/UAS-CHCHD2 T61I/+ (hCHCHD2^{T61I})

Fig. 6B
w^{1118}/Y; UAS-mitoGFP/UAS-LacZ; D42-GAL4/UAS-CHCHD2 WT (WT)
w^{1118}/Y; UAS-mitoGFP/UAS-LacZ; D42-GAL4/UAS-CHCHD2 P14L (P14L)
w^{1118}/Y; UAS-mitoGFP/UAS-LacZ; D42-GAL4/UAS-CHCHD2 T61I (T61I)

Fig. 6C
(Upper graph)

CG5010^{null}/Y; UAS-GCaMP6f/+; R58E02-Gal4/UAS-CHCHD2 WT (WT)
CG5010^{null}/Y; UAS-GCaMP6f/+; R58E02-Gal4/UAS-CHCHD2 P14L (P14L)
CG5010^{null}/Y; UAS-GCaMP6f/+; R58E02-Gal4/UAS-CHCHD2 T61I (T61I)

(Lower graph)
CG5010^{null}/Y; +/+; R58E02-Gal4, UAS-mito-GCaMP6/UAS-CHCHD2 WT (WT)
CG5010^{null}/Y; +/+; R58E02-Gal4, UAS-mito-GCaMP6/UAS-CHCHD2 P14L (P14L)
CG5010^{null}/Y; +/+; R58E02-Gal4, UAS-mito-GCaMP6/UAS-CHCHD2 T61I (T61I)

Fig. S8A-D, Fig. S8, S9
w^{1118} (TBPH$^{+/+}$)
TBPH^{G2}/TBPH^{G2} (TBPH$^{-/-}$)
w^{1118}/w^{1118} (dCHCHD2$^{+/+}$)
w^{1118}, CG5010^{null}/w^{1118}, CG5010^{null} (dCHCHD2$^{-/-}$)
w^{1118}; GMR-GAL4/UAS-TBPH-FLAG (TBPH-FLAG)

Fig. S10
w^{1118}, CG5010^{null}/Y; UAS-mitoGFP/+; Ddc-GAL4/UAS-CHCHD2 WT (WT)
w^{1118}, CG5010^{null}/Y; UAS-mitoGFP/+; Ddc-GAL4/UAS-CHCHD2 P14L (P14L)
w^{1118}, CG5010^{null}/Y; UAS-mitoGFP/+; Ddc-GAL4/UAS-CHCHD2 T61I (T61I)

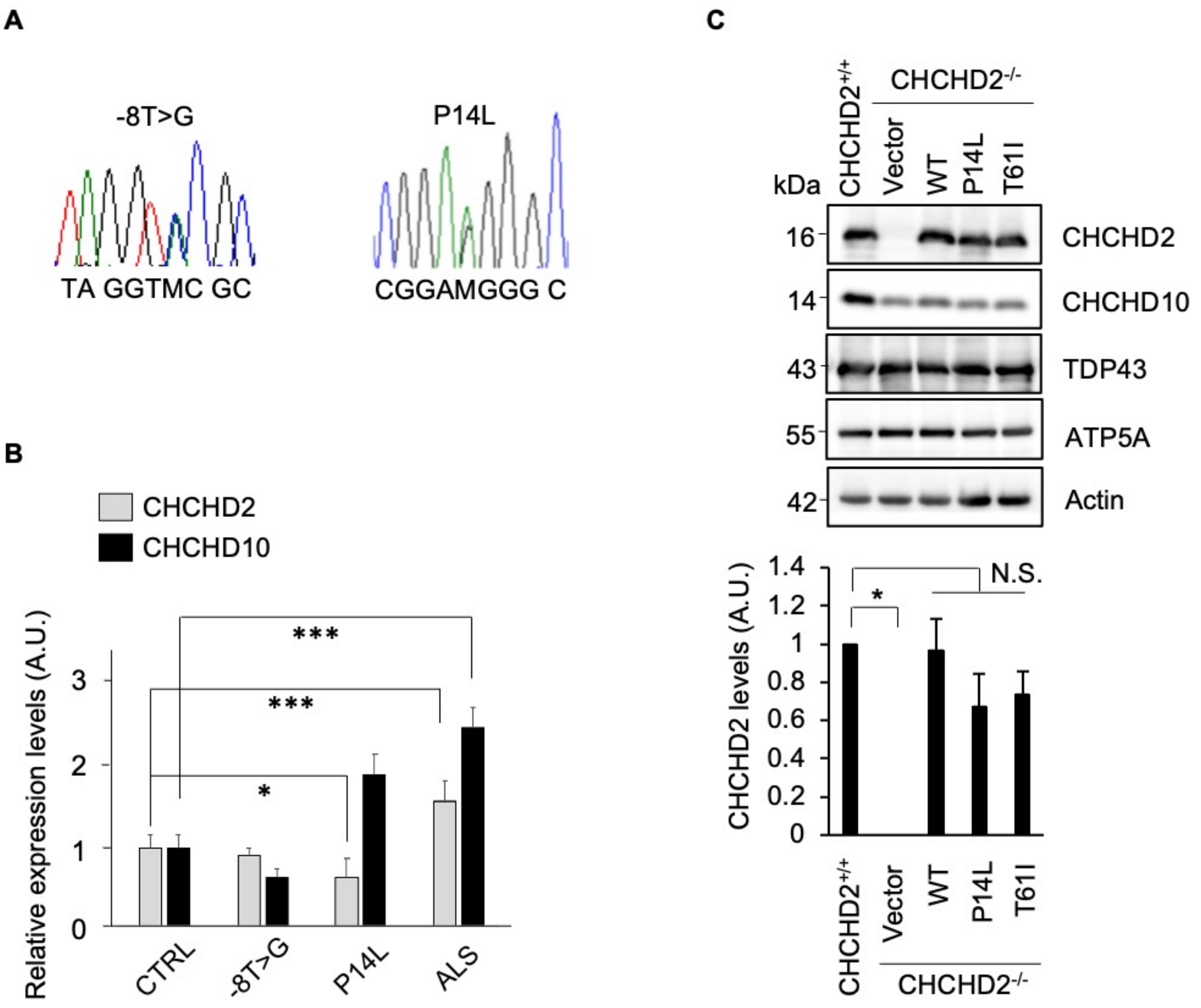

Figure S1. CHCHD2 is downregulated at the transcript level in the patient with CHCHD2 P14L. (A) Electropherogram of the Sanger sequence showing -8T>G and c.41C>T (P14L) in the *CHCHD2* gene. (B) The patient with CHCHD2 P14L showed reduced expression of *CHCHD2* transcripts. The graph shows the relative values of *CHCHD2* and *CHCHD10* transcripts normalized to *actin* transcripts (mean ± SEM, n = 4 technical replicates with three controls [CTRL], two ALS cases with CHCHD2 variants, and three sporadic ALS cases). The values of the CTRL and sporadic ALS groups are shown as aggregates. Transcript levels were determined by quantitative reverse transcription-PCR using total RNA extracted from the frontal lobes. *$p < 0.024$, ***$p < 0.001$ by Dunnett's test. (C) The compensatory expression of CHCHD2 in CHCHD2$^{-/-}$ SH-SY5Y cells was comparable to its endogenous level. Exogenous CHCHD2 variants were virally introduced, and the expression of CHCHD2, CHCHD10, TDP-43, and ATP5A was analyzed. Actin served as a loading control. The graph shows the relative values of CHCHD2 protein normalized to those of Actin (mean ± SEM, n = 3 biological replicates). *$p < 0.0005$ by Dunnett's test. A.U., arbitrary unit.

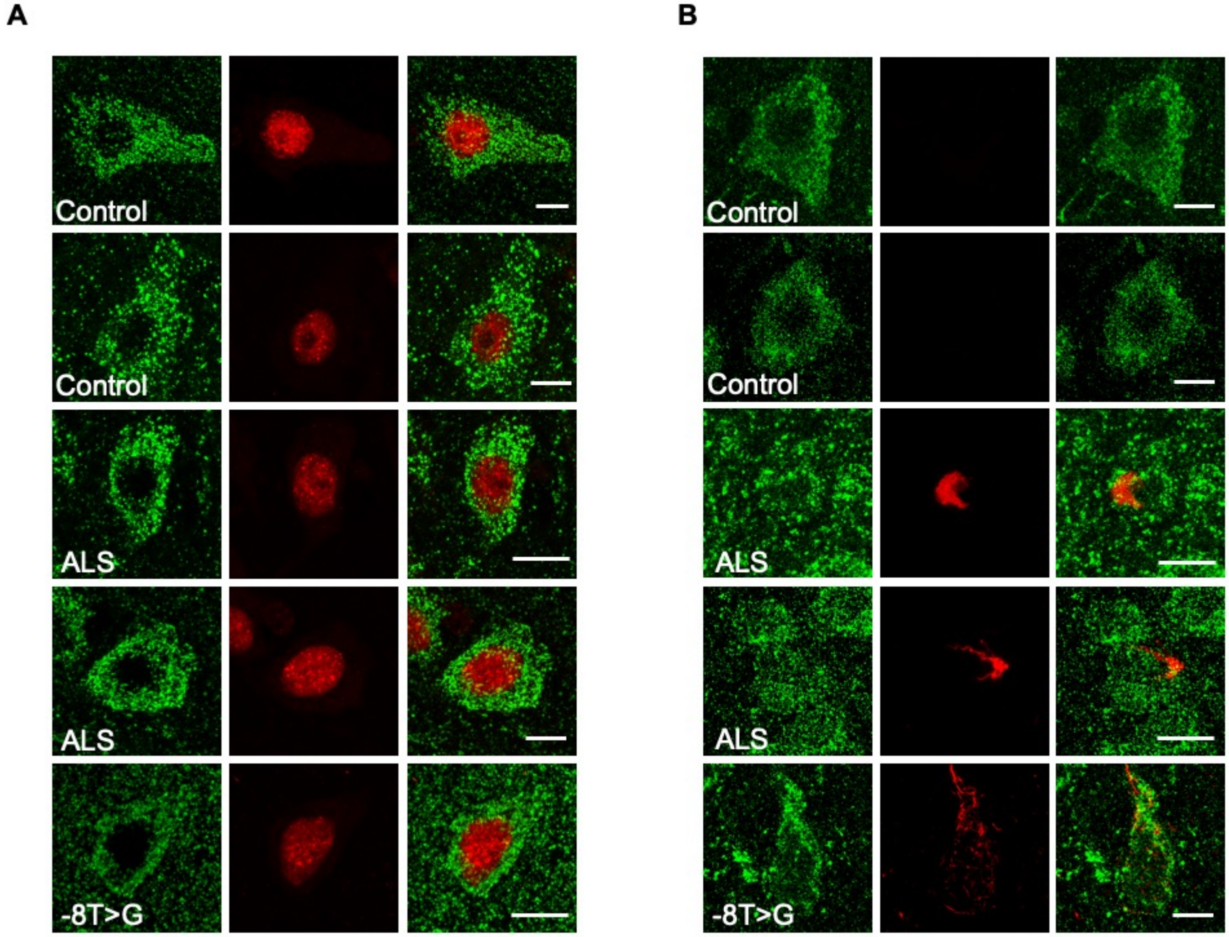

Figure S2. TDP-43 pathology in patients with ALS carrying CHCHD2 variants.
(A) Subcellular localization of ATP5A (green) and TDP-43 (red) in Betz cells in the primary motor cortex of normal controls, patients with sporadic ALS (ALS), and a patient with ALS carrying *CHCHD2* -8T>G. ATP5A served as a mitochondrial marker. (B) Subcellular localization of CHCHD2 (green) and phospho-TDP-43-positive inclusions (red) in Betz cells. Scale bars, 10 μm.

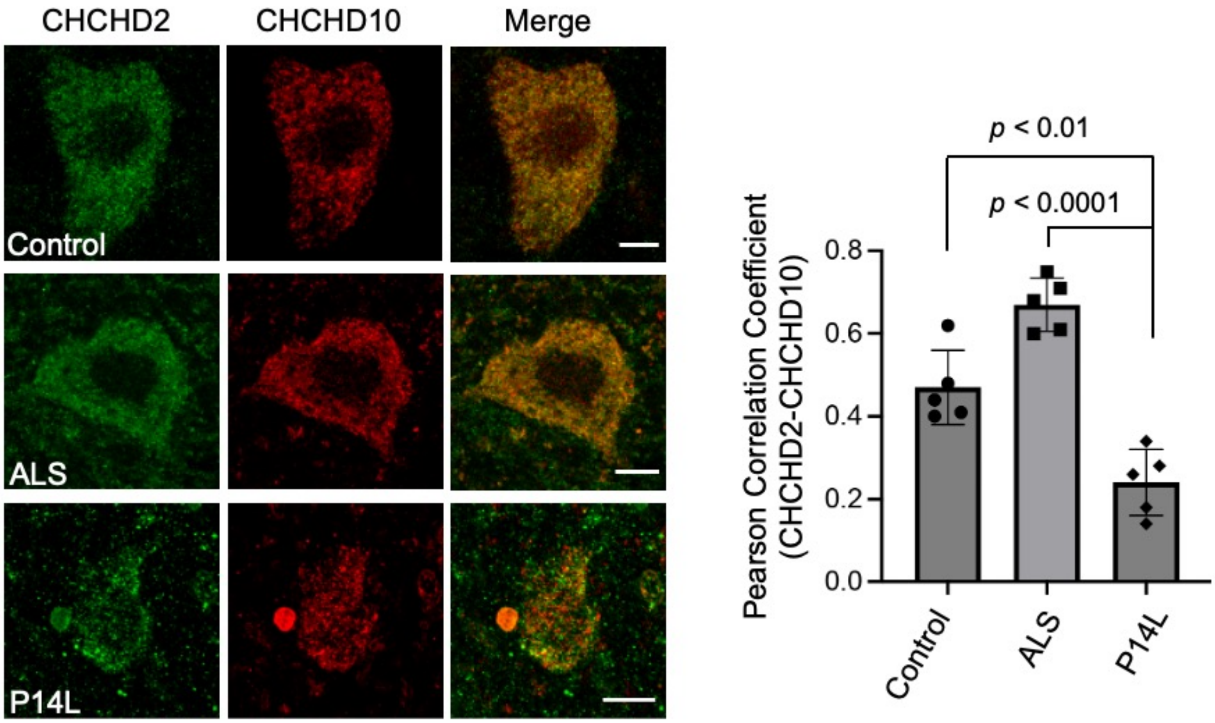

Figure S3. Subcellular localization of CHCHD2 and CHCHD10 in patients with ALS.
Localization of CHCHD2 and CHCHD10 in motor neurons of the anterior horn of the spinal cord. CHCHD2 P14L showed a loss of colocalization with CHCHD10, while CHCHD2 largely colocalized with CHCHD10 in patients with sporadic ALS (ALS) as well as in normal controls. Scale bars, 10 μm. The graph shows Pearson's correlation coefficients between CHCHD2 and CHCHD10 on the results as shown on the left. Comparison was determined by Tukey–Kramer's test. n = 5 cells in each group from autopsies of two controls, two patients with sporadic ALS, and one patient with ALS carrying the CHCHD2 P14L variant.

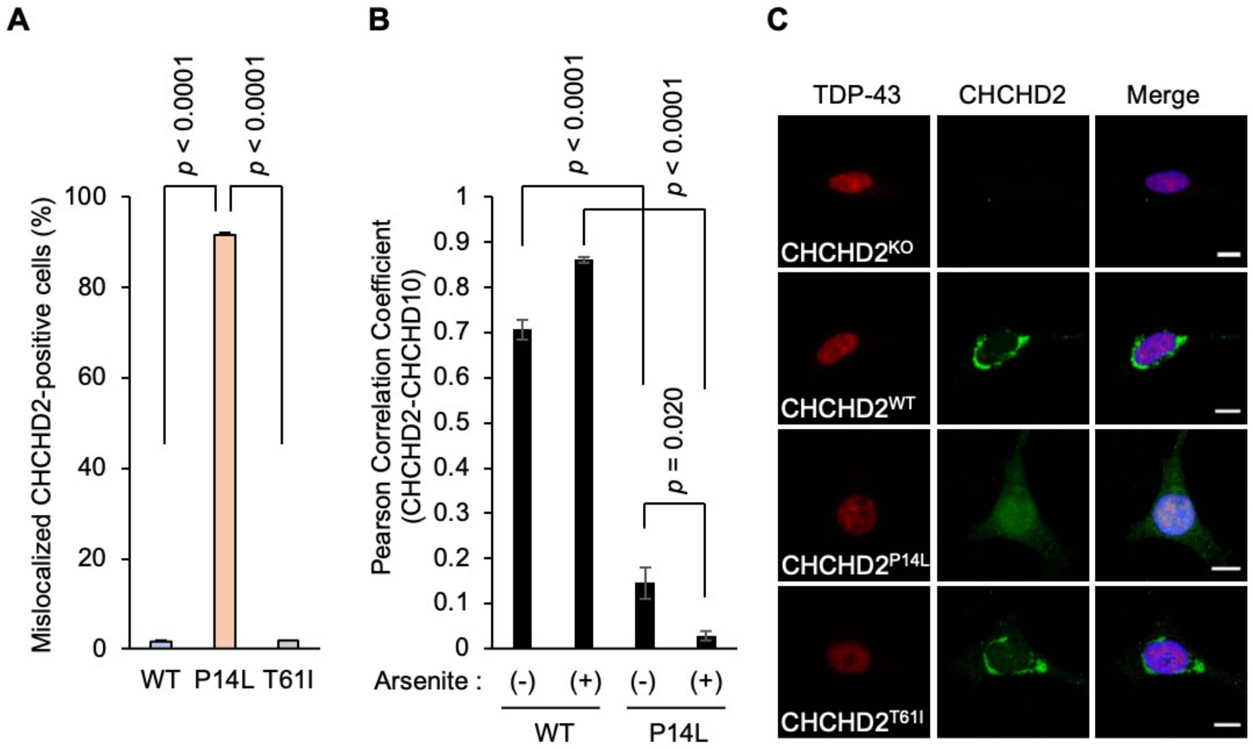

Figure S4. Subcellular localization of TDP-43 and CHCHD2 after arsenite treatment.

(A) Percentages (mean ± SEM) of cytosolic CHCHD2-positive cells under a steady-state condition as in Fig. 3B. Comparison was determined by Tukey–Kramer's test (more than 600 cells from 3 biological replicates). (B) Pearson's correlation coefficients between CHCHD2 and CHCHD10 in cells treated with or without 0.5 mM arsenite for 1 h as in Fig. 3D. Comparison was determined by Tukey–Kramer's test (more than 40 cells from 3 biological replicates). (C) $CHCHD2^{-/-}$ SH-SY5Y cells harboring the mock vector (KO), CHCHD2 WT, P14L, or T61I were treated with or without arsenite (0.5 mM) for 1 h. The CHCHD2 and TDP-43 signals were detected using specific antibodies. Scale bars, 10 µm.

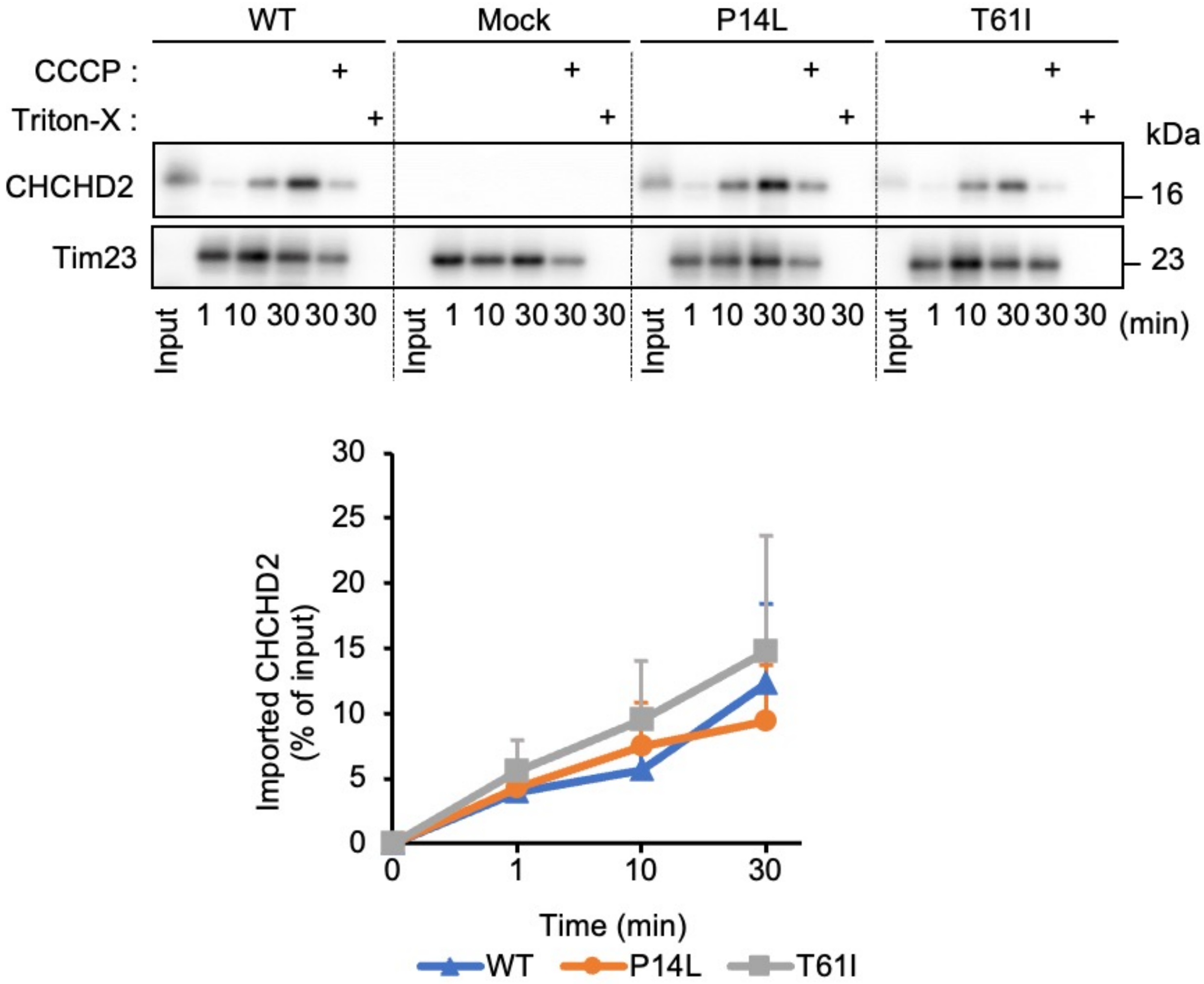

Figure S5. Mitochondrial import of CHCHD2 is not affected by P14L.
Mitochondrial import assay of CHCHD2 prepared by *in vitro* translation. Newly synthesized CHCHD2 in reticulocyte lysate was incubated with mitochondria (20 µg each) isolated from CHCHD2$^{-/-}$ SH-SY5Y cells for the indicated periods of time. After proteinase K treatment (20 µg/ml at a final concentration) to remove CHCHD2 outside mitochondria, CHCHD2 imported into the mitochondria was detected using an anti-CHCHD2 antibody. A reduction in CHCHD2 signals with 200 µM CCCP treatment indicated that the mitochondrial import of CHCHD2 is dependent on the mitochondrial membrane potential. The disappearance of CHCHD2 upon proteinase K treatment in the presence of 0.5% Triton X-100 confirmed the presence of CHCHD2 in mitochondria. The 10% input for each CHCHD2 variant is also shown. Tim23 served as a loading control for the mitochondria. The graph shows the intensity of the CHCHD2 band at the indicated time points.

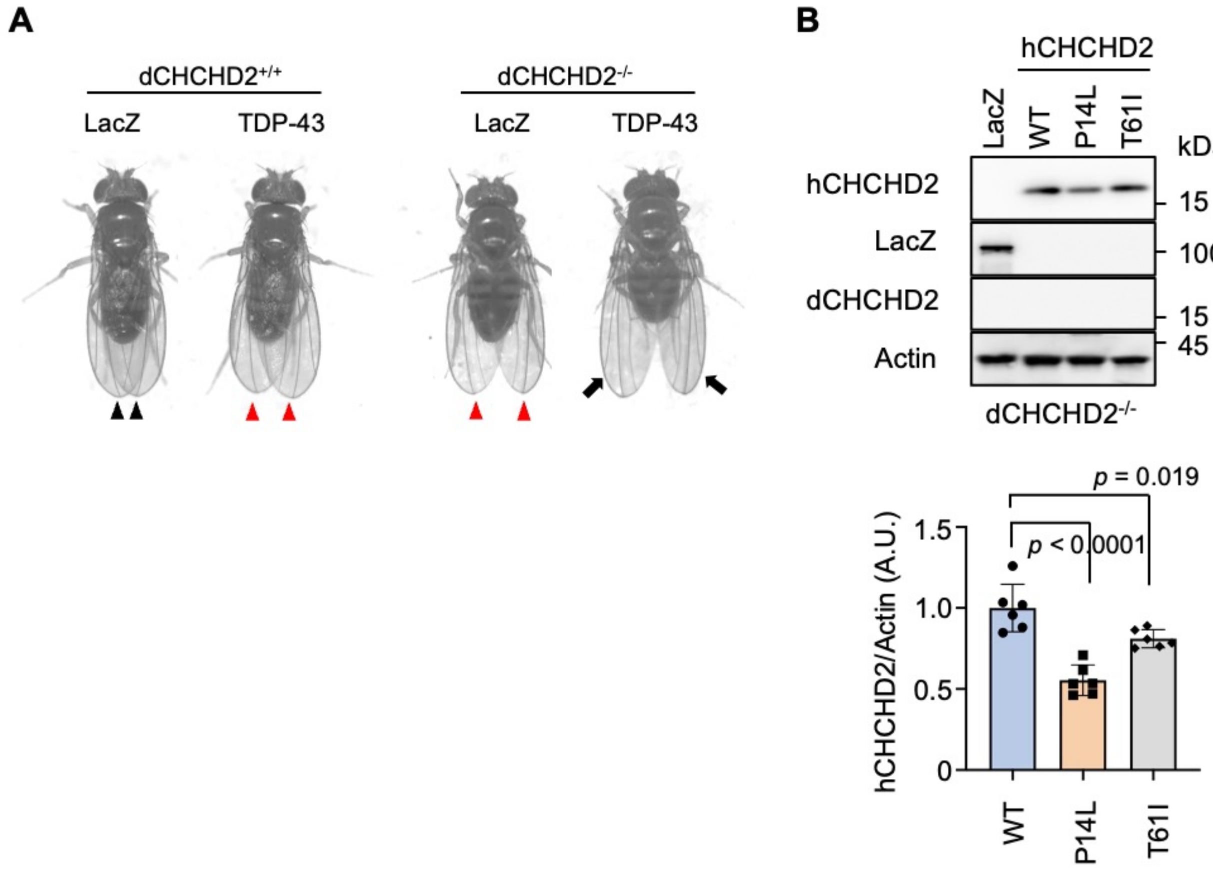

Figure S6. Muscular expression of TDP-43 exhibits wing phenotypes in the absence of dCHCHD2. (A) Muscular expression of human TDP-43 produced abnormal wing postures. *dCHCHD2*$^{+/+}$ flies expressing LacZ exhibited normal wing postures (black arrowheads), whereas *dCHCHD2*$^{+/+}$ flies expressing TBPH and *dCHCHD2*$^{-/-}$ flies expressing LacZ exhibited a flaring wing phenotype (red arrowheads). *dCHCHD2*$^{-/-}$ flies expressing TBPH exhibited a flaring and drooping wing phenotype (arrows). Transgenes were expressed by *MHC-GAL4* at 18 °C. Twenty-one-day-old flies were analyzed. (B) The protein expression levels of human CHCHD2 WT, P14L, and T61I were comparable in *dCHCHD2*-deficient flies. Protein expression was analyzed in the thoraces. Actin served as a loading control. The graph shows the relative values of hCHCHD2 protein normalized to those of Actin (mean ± SEM). Comparison was determined by Dunnett's test from 6 biological replicates. The expression levels of CHCHD2 P14L and T61I were reduced compared to those of WT, consistent with the findings in the human brain. This reduction in expression is likely attributable to protein instability.

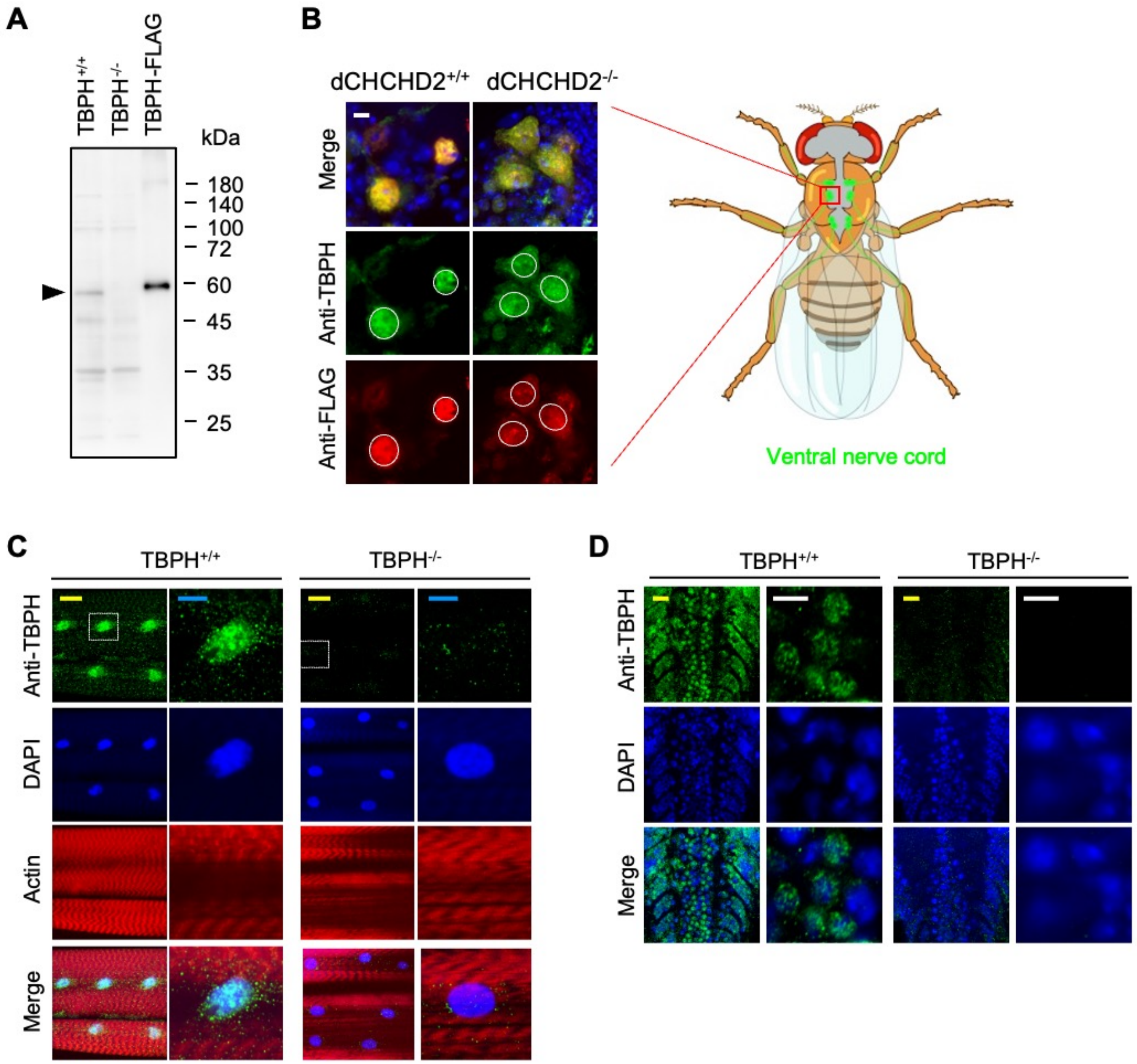

Figure S7. Generation of anti-TBPH antibody.
(A) Endogenous TBPH in 3rd-instar larvae was detected with newly generated rabbit anti-TBPH (1:200 dilution) in normal but not *TBPH*$^{-/-}$ flies. A 10x dilution of brain lysates expressing TBPH-FLAG by *GMR-GAL4* served as a positive control. *TBPH*^{G2} was used for the *TBPH* null allele (6). (B) The anti-TBPH antibody detected TBPH-FLAG expressed in motor neurons of the thoracic ganglion (the location is shown in the illustration). Circles indicate nuclear regions. The TBPH was localized outside of the nucleus in *dCHCHD2*$^{-/-}$ flies. Scale bar, 5 µm. (C, D) Endogenous TBPH is mainly localized in the nuclei of thoracic muscular cells (C) and neurons of the thoracic ganglion (D). Enlarged images of the dashed boxes are shown in the right columns (C). Yellow, blue, and white scale bars, 25 µm, 10 µm, and 5 µm, respectively.

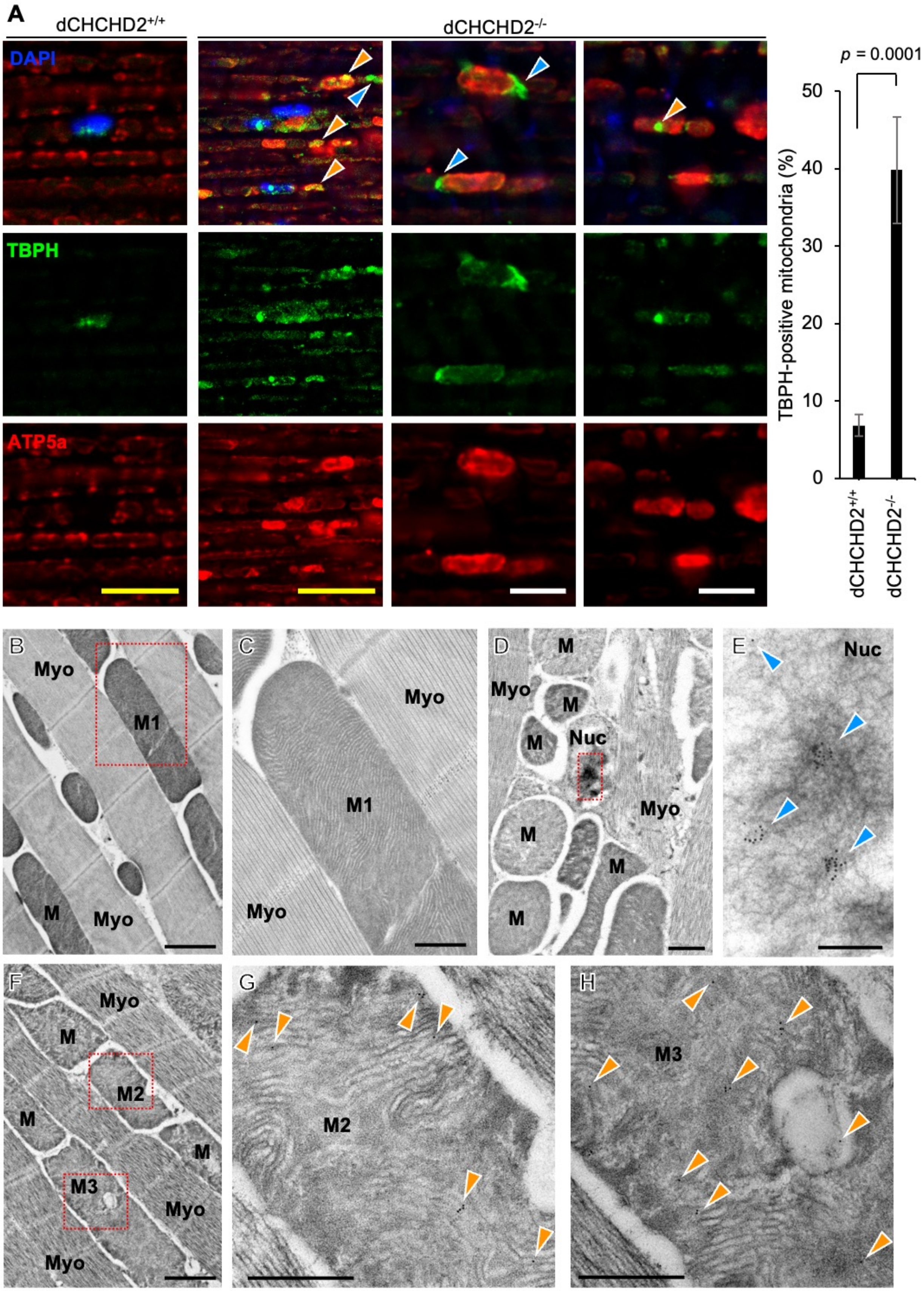

A
dCHCHD2+/+
dCHCHD2-/-
DAPI
TBPH
ATP5a
p = 0.0001
TBPH-positive mitochondria (%)
50
40
30
20
10
0
dCHCHD2+/+
dCHCHD2-/-
B
Myo
M1
M
Myo
C
Myo
M1
Myo
D
M
Myo
M
M
Nuc
Myo
M
M
M
E
Nuc
F
Myo
M
M2
M
M
M3
Myo
Myo
G
M2
H
M3

Figure S8. Mitochondrial localization of TBPH following dCHCHD2 loss.
(A) Subcellular localization of endogenous TBPH in the muscle cells of 30-day-old flies. TBPH, mitochondria, and nuclei were visualized by anti-TBPH, anti-ATP5a, and DAPI staining, respectively. Merged images and single-channel images for the indicated proteins are shown. The orange and blue arrowheads indicate mitochondrial TBPH and perimitochondrial TBPH signals, respectively. Yellow and white scale bars, 20 μm and 5 μm, respectively. The graph shows the total percentage (mean ± SEM) of mitochondria exhibiting TBPH signaling and of mitochondria where TBPH aggregations are localized at the periphery. Comparison was determined by two-tailed Student *t* test (n = 12 images from 3 flies for each genotype). (B-H) Immunoelectron microscopic analysis of TBPH labeled with 10-nm gold particles in the muscles of 30-day-old flies. Endogenous TBPH signals were mainly localized in the nucleus (blue arrowheads in E) but not in the mitochondria (B and C) in normal flies. (C and E) Enlarged images of the dashed red boxes in B and D, respectively. (F–H) TBPH signals were detected in the mitochondria (orange arrowheads in G and H) of *dCHCHD2*-deficient flies. (G and H) Enlarged images of the dashed red boxes in (F). M, mitochondrion; Myo, myofibrils; Nuc, nucleus. Scale bars, 2 μm in (B and F), 1 μm in (D), 500 nm in (C, G and H), and 200 nm in (E).

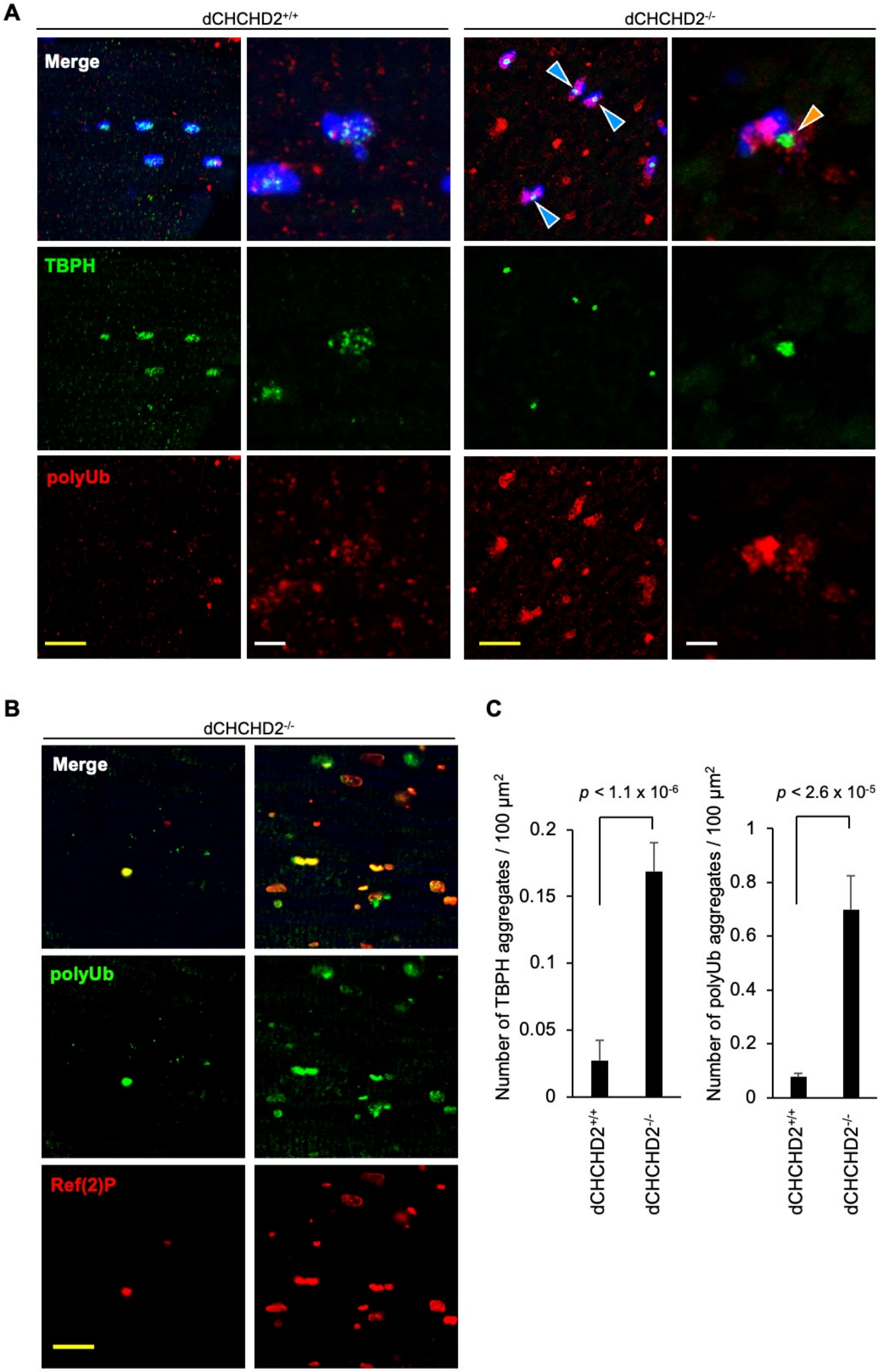
A
dCHCHD2+/+
dCHCHD2-/-
Merge
TBPH
polyUb
B
dCHCHD2-/-
Merge
polyUb
Ref(2)P
C
Number of TBPH aggregates / 100 µm2
p < 1.1 x 10-6
0.2
0.15
0.1
0.05
0
dCHCHD2+/+
dCHCHD2-/-
Number of polyUb aggregates / 100 µm2
p < 2.6 x 10-5
1
0.8
0.6
0.4
0.2
0
dCHCHD2+/+
dCHCHD2-/-

Figure S9. Polyubiquitin and fly p62/SQSTM1 Ref(2)P accumulate along with TBPH following dCHCHD2 loss.

(A) Polyubiquitin (polyUb) signals accumulated along with aggregated TBPH both within and outside of the thoracic muscle cell nuclei in *dCHCHD2*$^{-/-}$ flies. Thirty-day-old flies were analyzed. TBPH (green), polyUb (red), and nuclei (blue) were visualized by anti-TBPH, anti-polyUb, and DAPI staining, respectively. Merged images and single-channel images for the indicated proteins are shown. Blue and orange arrowheads indicate aggregated TBPH in the nuclei and perinuclear aggregated TBPH, respectively. Yellow and white scale bars, 10 µm and 2.5 µm, respectively. (B) Most polyUb signals were Ref(2)P-positive in the thorax muscles of 30-day-old *dCHCHD2*-deficient flies. Yellow scale bar, 10 µm. (C) The left graph presents the number (mean ± SEM) of TBPH aggregates larger than 0.35 μm^2 within a 100 µm² area. Comparison was determined by two-tailed Student *t* test (n = 9 images from 4-5 flies for each genotype). The right graph shows the number (mean ± SEM) of polyUb-positive aggregates larger than 1.5 μm^2 within a 100 µm² area. Comparison was determined by two-tailed Student *t* test (n = 10 images from 5 flies for each genotype).

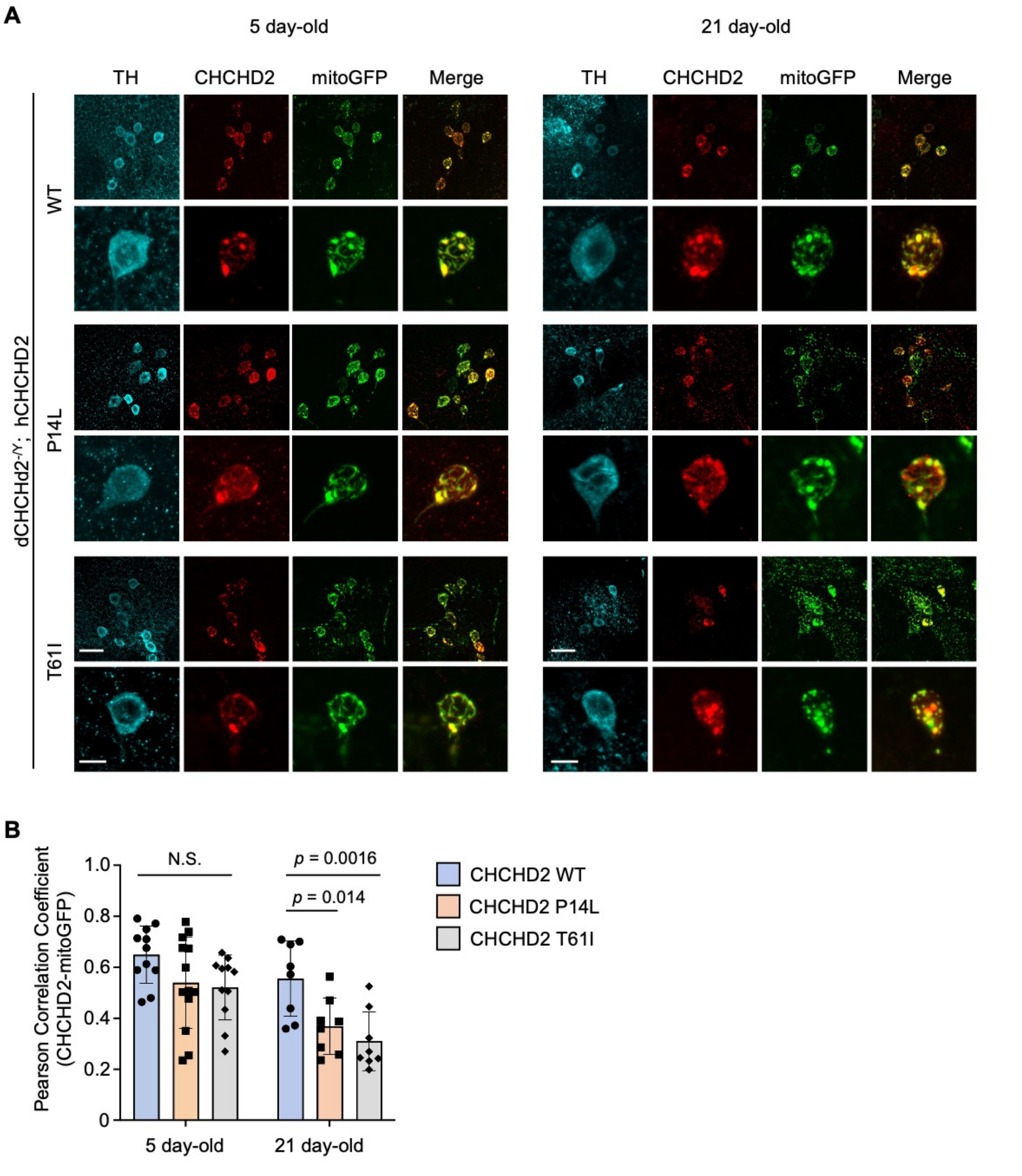

Figure S10. CHCHD2 mutations cause CHCHD2 mislocalization with age.
(A) Subcellular localization of human CHCHD2 in the dopaminergic neurons of 5-day-old and 21-day-old flies. Mitochondria and dopaminergic neurons were visualized by mitoGFP and anti-TH staining, respectively. Single-channel images for the indicated proteins and merged images of mitoGFP and CHCHD2 are shown. Scale bars, 20 (upper) and 5 (lower) μm. (B) The graph shows Pearson's correlation coefficients between CHCHD2 and mitoGFP as indicated from experiments in (A). Comparison was determined by Dunnett's test (n = 8-13 cells from 6 independent flies).

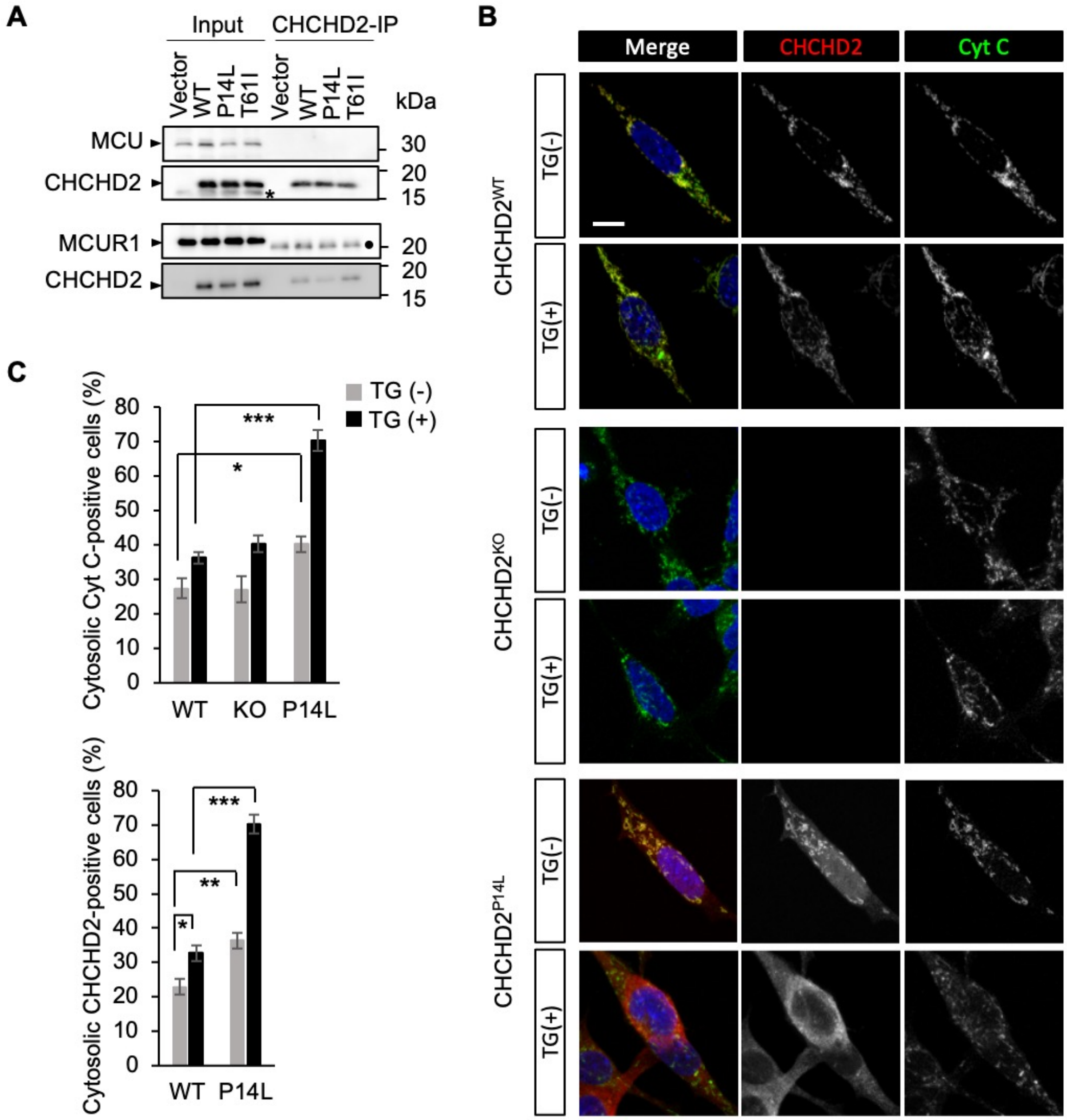

Figure S11. Cyt C and CHCHD2 release from mitochondria is accelerated by P14L upon cytoplasmic Ca^{2+} elevation.

(A) MCU and MCUR1 do not interact with CHCHD2. CHCHD2 was immunoprecipitated with an anti-CHCHD2 antibody in CHCHD2$^{-/-}$ SH-SY5Y cells harboring CHCHD2 variants or an empty vector, and coprecipitated proteins were analyzed with the indicated antibodies. The upper and lower panels show the results of independent trials. Asterisks and dots indicate endogenous CHCHD10 and anti-CHCHD2 antibody-derived signals, respectively. (B) SH-SY5Y cells stably expressing CHCHD2 variants or empty vector (KO) treated with 100 nM thapsigargin (TG +) or DMSO (TG -) for 24 h in a galactose-containing medium were stained with anti-cyt C (green) and anti-CHCHD2 (red). Nuclei were counterstained with DAPI (blue) in merged images. Scale bar, 10 μm. (C) Percentages (mean ± SEM) of cytosolic cyt C- and CHCHD2-positive cells after treatment with 100 nM thapsigargin (TG +) or DMSO (TG -) for 24 h. Upper graph, *p < 0.024, ***p < 0.0001 by Dunnett's test. (more than 900 cells from 3 biological replicates). Lower graph, *p < 0.020, **p < 0.0040, ***p < 0.0001 by two-tailed Student t test (more than 900 cells from 3 biological replicates).

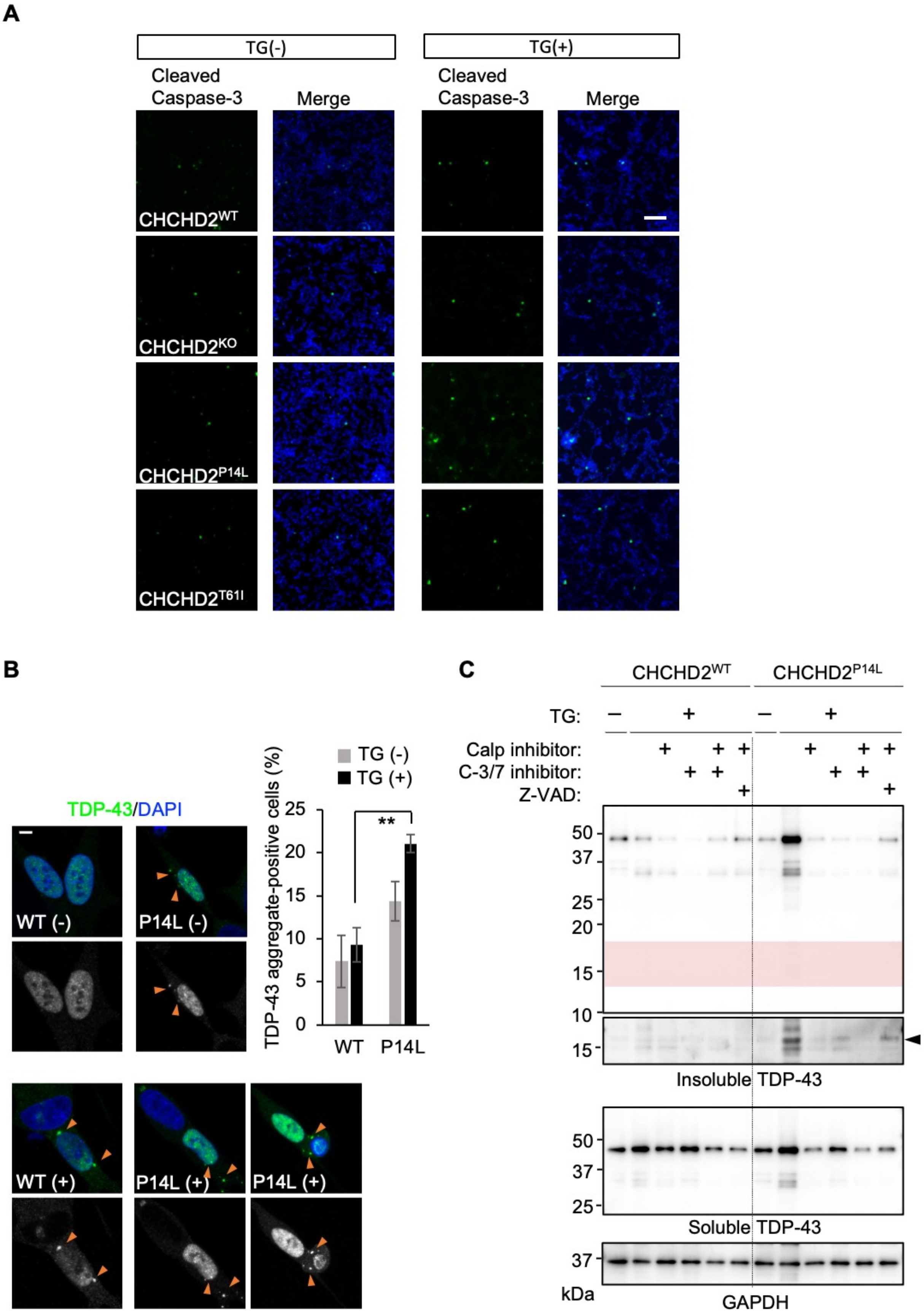

A
TG(-)
TG(+)
Cleaved Caspase-3
Merge
CHCHD2WT
CHCHD2KO
CHCHD2P14L
CHCHD2T61I
B
TDP-43/DAPI
WT (-)
P14L (-)
WT (+)
P14L (+)
TG (-)
TG (+)
TDP-43 aggregate-positive cells (%)
25
20
15
10
5
0
**
WT
P14L
C
CHCHD2WT
CHCHD2P14L
TG:
Calp inhibitor:
C-3/7 inhibitor:
Z-VAD:
50
37
25
20
15
10
Insoluble TDP-43
Soluble TDP-43
GAPDH
kDa

Figure S12. Insoluble C-terminal fragments of TDP-43 are generated by calpain and caspases. (A) CHCHD2$^{-/-}$ SH-SY5Y cells stably expressing CHCHD2 variants or empty vector (KO) treated with 100 nM thapsigargin (TG +) or DMSO (TG -) for 24 h in a galactose-containing medium were stained with an anti-cleaved caspase-3 antibody (green). Nuclei were counterstained with DAPI (blue) in merged images. Scale bar, 100 μm. (B) TDP-43 aggregation is increased in CHCHD2$^{-/-}$ SH-SY5Y cells stably expressing CHCHD2 P14L by thapsigargin treatment as in (A). **p < 0.0054 by two-tailed Student t test (more than 300 cells from 3 biological replicates). Representative aggregates with (+) or without (-) thapsigargin treatment are indicated by arrowheads in images, which were visualized with anti-TDP-43 antibody (green in the merged and greyscale images). Scale bar, 5 μm. Note that the phosphorylated TDP-43 signals were not adequately detected. The formation of phosphorylated TDP-43 aggregates is likely to require an extended period to replicate chronic pathological conditions. (C) Calpain- and caspase-dependent generation of insoluble C-terminal fragments of TDP-43. CHCHD2$^{-/-}$ SH-SY5Y cells stably expressing CHCHD2 WT or P14L were pretreated with or without 10 μM calpain inhibitor I (Calp inhibitor), 20 μM pancaspase inhibitor z-VAD-fmk (z-VAD), and/or 50 μM caspase-3/7 inhibitor (C-3/7 inhibitor) for 1 h before treatment with 100 nM thapsigargin (TG +) or DMSO (TG -) for 24 h in a galactose-containing medium. TDP-43 was fractionated with 1% Triton X-100-containing buffer to obtain soluble and insoluble TDP-43. A long-exposed image of the region highlighted in red in the insoluble TDP-43 blot, which detects the ~17-kDa insoluble TDP-43 C-terminal fragment (arrowhead), is also shown.

Table S1. Frequency of the *CHCHD2* rare variants.

Detected rare variants				Allele frequencies			JaCALS *vs.* jMorp		JaCALS *vs.* gnomAD east Asian	
	cDNA	Amino acid	rs number	JaCALS (N=944)	JMorp (N=54,302)	gnomAD Eeat Asian (N=44,850)	OR (95% CI)	*P* value	OR (95% CI)	*P* value
chr7:56174114	–8T>G	5'UTR	rs1584645975	0.00053	0.000138	0.000112	7.67 (1.01-58.12)	0.0197	4.75 (0.56-40.69)	0.116
chr7:56174066	41C>T	Pro14Leu	rs776234231	0.00053	0.000166	0.0000446	6.39 (0.85-47.92)	0.0379	11.88 (1.08-131.01)	0.00998

The allele frequencies of the variants between the JaCALS cohort and a general Japanese population were compared using the χ^2 test.
CI, confidence interval; gnomAD, the genome aggregation database, JaCALS, Japanese Consortium for Amyotrophic Lateral Sclerosis Research; jMorp, Japanese Multi-Omics Reference Panel; OR, odds ratio; rs number, Reference SNP ID number.

Table S2. Human brain tissue samples.

	Sex	Age at onset (years)	Disease duration (years)	Age at death	PMI (h)	Dementia	Parkinson-ism	Neuropathology
**CTRL1**	F	N/A	N/A	77	2	-	-	Normal
**CTRL2**	M	N/A	N/A	72	86	-	-	Normal
**CTRL3**	M	N/A	N/A	86	1	-	-	Normal
**ALS1**	F	70	3	73	4.5	-	-	TDP-43
**ALS2**	M	81	1.5	82	3.5	+	-	TDP-43
**ALS3**	M	67	2	69	2.5	-	-	TDP-43
**ALS4**	M	70	1.5	72	3	-	-	TDP-43
**ALS5**	M	63	8	71	6	-	-	TDP-43
**CHCHD2 -8T>G**	M	66	6	72	4.7	-	-	TDP-43
**CHCHD2 P14L**	M	23	22	45	12	-	-	TDP-43
**CHCHD2 T61I**	F	49	13	62	N/A	-	+	Synucleinopathy

CTRL, control; M, male; F, female; PMI, postmortem interval; N/A, not available.

Table S3. Primary antibodies utilized in this study.

Targets	Host	Clone	Dilution	Application	Source
**CHCHD2**	Rabbit	Polyclonal	1:200	IHC (Human tissues)	Proteintech (19424-1-AP)
**CHCHD10**	Mouse	11F11.2	1:200	IHC (Human tissues)	Merck (MABN1524)
**ATP5A**	Mouse	15H4C4	1:1000	IHC (Human tissues)	Abcam (ab14748)
**TDP-43**	Rabbit	Polyclonal	1:200	IHC (Human tissues)	Proteintech (10782-2-AP)
**pTDP-43 (pS409/410)**	Mouse	11-9	1:3000	IHC (Human tissues)	Cosmo Bio (TIP-PTD-M01)
**CHCHD2**	Rabbit	Polyclonal (R2)	1:2000	WB	In-house (7)
**CHCHD2**	Rabbit	Polyclonal	1/500	ICC	Proteintech (19424-1-AP)
**CHCHD10**	Mouse	11F11.2	1:1000, 1/500	WB, ICC	Merck (MABN1524)
**TDP-43**	Rabbit	Polyclonal	1:1000, 1/500	WB, ICC	Proteintech (10782-2-AP)
**TDP-43**	Mouse	3H8	1:1000, 1/500	WB, ICC	Abcam (ab104223)
**pTDP-43 (pS409/410)**	Mouse	11-9	1:1000	WB	Cosmo Bio (TIP-PTD-M01)
**Tom20**	Mouse	Polyclonal	1:1000, 1/200	WB, ICC	BD (612278)
**ATP5A**	Mouse	15H4C4	1:2000	WB (Human tissues)	Abcam (ab14748)
**Tim23**	Mouse	32/Tim23	1:1000	WB (Human tissues)	BD (611223)
**p62/SQSTM1**	Rabbit	Polyclonal	1:1000	WB (Human tissues)	MBL (PM045)
**LDHA**	Rabbit	Polyclonal	1:1000	WB	Abcam (ab47010)
**Mitofusin1**	Mouse	3C9	1:1000	WB	Abnova (H00055669)
**PARP**	Rabbit	N2C1	1:1000	WB	GeneTex (GTX112864)
**MCU**	Rabbit	D2Z3B	1:1000	WB	Cell Signaling Technology (#14997)
**MCUR1**	Rabbit	Polyclonal	1:1000	WB	Cell Signaling Technology (#13706)
**Cleaved caspase-3**	Rabbit	D175	1:200	ICC	Cell Signaling Technology (#9661)
**Cyt C**	Mouse	6H2B4	1:200	ICC	BD (556432)
**Actin**	Mouse	C4	1:1000	WB (Human tissues)	Merck (MAb1501)
**dCHCHD2**	Rabbit	Polyclonal	1:1000	WB (Drosophila)	In-house (7)
**LacZ (β-galactosidase)**	Mouse	Z3781	1:1000	WB (Drosophila)	Promega (Z3781)
**Ref(2)P**	Rabbit	Polyclonal (1532052)	1:100	WB (Drosophila)	In-house (3)
**ATP5A**	Mouse	15H4C4	1:20000, 1:500	WB, IHC (Drosophila)	Abcam (ab14748)
**TBPH**	Rabbit	Polyclonal	1:200, 1:50, 1:50	WB, IHC with Can get signal Solution A, IM (Drosophila)	In-house, made for this study

**FLAG**	Mouse	M2	1:1000, 1:200	WB, IHC (Drosophila)	Merck (F1804）
**CHCHD2**	Rabbit	Polyclonal	1:300	IHC (Drosophila)	Proteintech (19424-1-AP)
**Polyubiquitin**	Mouse	FK2	1:250	WB (Drosophila)	MBL (D058-3)
**Actin**	Mouse	C4	1:10000	WB (Drosophila)	Merck (MAb1501）
**Tyrosine hydroxylase**	Chicken	Polyclonal	1:500	IHC (Drosophila)	Abcam (ab76442)
**Tyrosine hydroxylase**	Rabbit	Polyclonal	1:500	IHC (Drosophila)	In-house (8)

IHC, immunohistochemistry; WB, western blot; ICC, immunocytochemistry; IM, immunoelectron microscopy.

SI References

1. Y. Naito, K. Hino, H. Bono, K. Ui-Tei, CRISPRdirect: software for designing CRISPR/Cas guide RNA with reduced off-target sites. *Bioinformatics* **31**, 1120-1123 (2015).

2. F. A. Ran *et al.*, Genome engineering using the CRISPR-Cas9 system. *Nat Protoc* **8**, 2281-2308 (2013).

3. A. Ikeda *et al.*, Mutations in CHCHD2 cause alpha-synuclein aggregation. *Human molecular genetics* **28**, 3895-3911 (2019).

4. M. Harsch, K. Bendrat, G. Hofmeier, D. Branscheid, A. Niendorf, A new method for histological microdissection utilizing an ultrasonically oscillating needle: demonstrated by differential mRNA expression in human lung carcinoma tissue. *Am J Pathol* **158**, 1985-1990 (2001).

5. L. M. Murschall, E. Peker, T. MacVicar, T. Langer, J. Riemer, Protein Import Assay into Mitochondria Isolated from Human Cells. *Bio Protoc* **11**, e4057 (2021).

6. D. J. Hazelett, J. C. Chang, D. L. Lakeland, D. B. Morton, Comparison of parallel high-throughput RNA sequencing between knockout of TDP-43 and its overexpression reveals primarily nonreciprocal and nonoverlapping gene expression changes in the central nervous system of Drosophila. *G3 (Bethesda)* **2**, 789-802 (2012).

7. H. Meng *et al.*, Loss of Parkinson's disease-associated protein CHCHD2 affects mitochondrial crista structure and destabilizes cytochrome c. *Nat Commun* **8**, 15500 (2017).

8. Y. Yang *et al.*, Mitochondrial pathology and muscle and dopaminergic neuron degeneration caused by inactivation of Drosophila Pink1 is rescued by Parkin. *Proceedings of the National Academy of Sciences of the United States of America* **103**, 10793-10798 (2006).
